# Clinical value of the new obesity definition for cardiovascular events in a Middle Eastern population: the TLGS

**DOI:** 10.1016/j.ajpc.2026.101766

**Published:** 2026-07-23

**Authors:** Soroush Masrouri, Parto Hadaegh, Navid Ebrahimi, Amir Abdi, Fereidoun Azizi, Farzad Hadaegh

**Affiliations:** a Prevention of Metabolic Disorders Research Center, Research Institute for Metabolic and Obesity Disorders, Research Institute for Endocrine Sciences, Shahid Beheshti University of Medical Sciences, Tehran, Iran; b Student Research Committee, School of Medicine, Tehran Medical Sciences, Islamic Azad University, Tehran, Iran; c Endocrine Research Center, Research Institute for Endocrine Sciences, Shahid Beheshti University of Medical Sciences, Tehran, Iran

**Keywords:** Obesity, Central obesity, Sex differences, Cardiovascular diseases

## Abstract

**Background:**

We assessed the sex-stratified association of the newly proposed *Lancet Commission* obesity definition with cardiovascular disease (CVD) in a Middle Eastern population.

**Methods:**

We analyzed 9429 Tehran Lipid and Glucose Study (TLGS) participants to assess obesity prevalence, and 7781 participants (mean age 46.8 years) were included in the longitudinal analysis for incidence of first CVD. Cox proportional hazards models estimated hazard ratios (HRs) and 95% confidence intervals (CIs) for associations of the new obesity definition and obesity phenotypes (i.e., anthropometric-only and BMI-plus-anthropometric obesity) with incident total CVD and CVD mortality.

**Results:**

Obesity prevalence was 67.2% (65.1% in men vs. 69.0% in women) under the new definition, compared with 26.3% (15.6%vs. 35.3%) under the traditional definition. Over a median follow-up of 19.9 years, 1450 participants developed CVD, and 344 died from CVD. Obesity showed stronger associations with CVD and CVD mortality in women than in men (*P*_interaction_ <0.01). After adjustment for sociodemographic and behavioral factors, HRs in women vs. men were 1.91 (95% CI, 1.49–2.44) vs. 1.48 (1.26–1.74) for CVD, and 2.98 (1.49–5.92) vs. 1.26 (0.92–1.74) for CVD mortality. HRs for anthropometric-only and BMI-plus-anthropometric obesity for CVD were 1.87 (1.43–2.43) and 1.95 (1.50–2.53) in women, and 1.45 (1.23–1.71) and 1.62 (1.31–2.00) in men. After adjustment for cardiometabolic risk factors and competing risks of non-CVD death, the new obesity definition remained associated with CVD in men and with both CVD and CVD mortality in women.

**Conclusions:**

The new definition substantially increased obesity prevalence and showed that anthropometric-only obesity carries excess CVD risk, especially among women.

## 1 Introduction

Obesity is a major global contributor to cardiovascular disease (CVD) and premature mortality. In addition to its rising prevalence, obesity is associated with a clustering of risk factors, including high blood pressure, dyslipidemia, and impaired glucose metabolism, which collectively accelerate atherosclerotic progression and lead to adverse cardiovascular outcomes [1,2]. According to recent Global Burden of Disease (GBD) data, high body mass index (BMI) accounted for an estimated 3.7 million deaths globally in 2021, of which approximately 1.9 million were attributable to CVD [3,4]. Iran, in particular, bears a substantial burden of obesity. In a 2024 umbrella systematic review, the prevalence of overweight and obesity among Iranian adults was estimated at approximately 27% and 17%, respectively. Notably, the prevalence of obesity was higher among women than men, at 18.6% and 12.7%, respectively [5].

In clinical and epidemiologic practice, obesity is still assessed using BMI; although straightforward, it is an imperfect surrogate for adiposity because it does not distinguish between fat and lean mass or account for body fat distribution [6]. Consequently, individuals with comparable BMI may display significantly divergent cardiometabolic risk profiles, and BMI-based categorization may misclassify many at-risk individuals [7]. Emerging evidence indicates that integrating measures of central adiposity might yield a more accurate prediction of cardiovascular risk than BMI alone [2].

The *Lancet Diabetes & Endocrinology Commission* recently proposed a revised framework in which excess adiposity is confirmed by direct body-fat measurement, where available, or by at least one anthropometric criterion in addition to BMI, including waist circumference (WC), waist-hip ratio (WHR), and waist-height ratio (WHtR) [8]. Under this framework, obesity may be defined by [1] elevated BMI (≥30 kg/m²) plus at least one abnormal anthropometric measure, or by BMI ≥40 kg/m², or by [2] at least two abnormal anthropometric measures in the absence of elevated BMI [8]. This revised approach may substantially influence the identification of obesity in clinical and population settings, with potential implications for patients, clinicians, and policymakers. Nevertheless, its prognostic significance and relevance to long-term health outcomes remain to be investigated in diverse populations. This gap is particularly relevant in Iran and the broader Middle East and North Africa (MENA) region, where obesity and cardiometabolic risk factors are highly prevalent, and population-based evaluations of this framework remain limited [9]; moreover, sex differences in adiposity distribution and its cardiovascular consequences make a sex-stratified evaluation particularly important [10,11].

In this study, within the framework of the Tehran Lipid and Glucose Study (TLGS), a well-characterized, community-based cohort with long-term follow-up, we sought to: (1) determine the prevalence of obesity based on the new definition compared to the conventional BMI-based approach, (2) assess their associations with incident CVD and its subtypes, and (3) explore potential sex-specific disparities in these associations.

## 2 Materials and methods

The TLGS is a large community-based prospective cohort study initiated in 1999 in district 13 of Tehran to investigate cardiometabolic risk factors in an urban population. In phase 1 from 1999 to 2001, 15,005 residents aged 3–69 years were enrolled through multistage stratified cluster random sampling and underwent baseline assessment using questionnaires, clinical examination, and laboratory testing. In phase 2, conducted from 2002 to 2005, the initially recruited participants were re-examined, and 3550 new individuals were enrolled, bringing the total number of participants to 18,555. Thereafter, follow-up examinations were performed at nearly 3-year intervals through phases 3 to 7, from 2006 to 2021. Further details of the study design and data collection have been published elsewhere [12]. The study was approved by the institutional review board of the Research Institute for Endocrine Sciences, Shahid Beheshti University of Medical Sciences, and written informed consent was obtained from all participants.

### 2.1 Study population

The analytic sample was derived from 9752 TLGS participants aged ≥30 years, as detailed in Figure S1, and was defined separately for the cross-sectional prevalence and longitudinal outcome analyses. For the cross-sectional prevalence analysis, 9429 individuals with available anthropometric measurements were included. For the longitudinal analysis, to focus on first incident CVD events, participants with a history of CVD at baseline (n = 604) were excluded, leaving 9148 individuals eligible for inclusion. After further excluding those with missing anthropometric measurements (n = 313), those without any follow-up data (n = 761), and those with missing covariate data (n = 293), 7781 participants remained for the longitudinal analysis. Participants with prevalent CVD at baseline were retained in the cross-sectional prevalence analysis, as the aim was to estimate the prevalence of obesity in the cohort population.

### 2.2 Clinical and laboratory measurements

During each phase, TLGS staff used standardized questionnaires to collect data on demographics, smoking status, family history of CVD, history of CVD, medication use, and education level. Anthropometric measurements included weight, height, WC, and hip circumference (HC) [12]. BMI was calculated as weight divided by height squared. Systolic blood pressure (SBP) and diastolic blood pressure (DBP) were measured twice, at 5‑minute intervals, on the right arm using a standardized protocol after the participant had remained seated at rest for 15 min, and the average of the two readings was used for analysis. For biochemical analyses, creatinine, fasting plasma glucose (FPG), and lipid profile (total cholesterol [TC], triglycerides [TG], and high-density lipoprotein cholesterol [HDL-C]) were measured from blood samples obtained in the morning after at least 12 h of overnight fasting. All obtained samples were analyzed on the day of blood collection at the TLGS research laboratory. Serum creatinine was measured using the kinetic colorimetric Jaffe method. Lipid levels were determined by an enzymatic colorimetric method. LDL-C was calculated using the Martin/Hopkins equation [13], which has been externally validated in populations within and outside the US [[14], [15], [16], [17], [18]]. The estimated glomerular filtration rate (eGFR) was calculated using the Chronic Kidney Disease Epidemiology Collaboration (CKD‑EPI) equation [19]. All analyses were performed using Pars Azmon kits (Pars Azmon Inc., Tehran, Iran) on a Selectra 2 auto‑analyzer (Vital Scientific, Spankeren, Netherlands), and samples were processed only after fulfilling internal quality control requirements. Assay quality was monitored after every 25 tests via lyophilized serum controls in normal and different abnormal concentrations.

### 2.3 Definition of terms

Family history of CVD was defined as the presence of coronary heart disease (CHD) or stroke in a first-degree relative before the age of 55 years (before 65 years for female relatives). Diabetes was defined as FPG ≥7.0 mmol/L (126 mg/dL) or use of anti-diabetic medications. CKD was defined as an eGFR <60 mL/min/1.73 m^2^. Hypertension was defined as SBP ≥140 mmHg or DBP ≥90 mmHg or use of anti-hypertensive medications. Dyslipidemia was defined as TC ≥240 mg/dL or LDL-C ≥ 160 mg/dL or TG ≥150 mg/dL or HDL-C < 40 mg/dL or use of lipid-lowering medications. Traditional obesity was defined as elevated BMI only, according to World Health Organization categories (obesity class I-III: ≥30 kg/m²).

In line with the new *Lancet Commission Obesity Framework* and previous studies [8,[20], [21], [22]], obesity phenotypes under the new definition were classified as follows:

1. BMI plus anthropometric obesity: BMI levels of 30–39.9 kg/m² plus ≥1 elevated anthropometric measure (i.e., WC ≥88 cm for women, ≥102 cm for men; WHR >0.85 for women, >0.90 for men; or WHtR ≥0.50), or BMI ≥40 kg/m² regardless of anthropometric measures;
2. Anthropometric-only obesity: ≥2 elevated anthropometric measures in the presence of a non-elevated BMI (<30 kg/m²).

In another analysis, based on Iranian population-specific thresholds for predicting CVD events, an elevated WC was defined as ≥95 cm for both sexes [23].

Cardiovascular endpoint definitions have been described elsewhere in detail [12,24]. Briefly, participants were followed annually to ascertain endpoints. Each year, trained nurses contacted TLGS participants via telephone to identify any medical events occurring during the previous year. If a possible event was reported, a trained physician collected complementary information through hospital or home visits and review of medical records. For deaths, data were obtained from death certificates, verbal autopsy, and forensic medicine reports [24]. Total CVD events included fatal and nonfatal CHD or stroke. CHD events comprised definite myocardial infarction (MI) confirmed by diagnostic electrocardiogram (ECG) findings and cardiac biomarkers [25]; probable MI based on positive ECG findings according to the American Heart Association’s (AHA’s) case-classification criteria [25] together with cardiac symptoms or signs in the absence of biomarker data, or with equivocal biomarker results; unstable angina pectoris; angiography-proven CHD (≥50% stenosis in ≥1 major coronary vessels); history of angioplasty or bypass surgery; and CHD death. For each outcome, only the first event was considered an incident. The event date for outcomes was defined with the exact date of the incidence of the event. Follow-up time was calculated as the difference between the study entrance time and the event date or censoring (loss to follow-up, non-cardiovascular mortality, or reaching the end of the study [2021]), whichever occurred first.

## 3 Statistical analysis

Analyses were performed using Stata version 14 SE (Stata Corp LP, College Station, TX, USA) and R version 4.4.0. Baseline characteristics are presented as mean (standard deviation) or median (interquartile range [IQR]) for continuous variables, according to their distribution, and as number (%) for categorical variables. Baseline characteristics were compared across the obesity-free, anthropometric-only obesity, and BMI-plus-anthropometric obesity groups, as well as between the two obesity groups, using chi-square tests, *t*-tests, ANOVA, Kruskal-Wallis tests, or Mann-Whitney U tests, as appropriate. For the cross-sectional analysis, the prevalence of obesity based on both the traditional and the new definition was estimated overall, by sex, and across prespecified age groups of young (<45 years), middle-aged (45 to 59 years), and older adults (≥60 years). Moreover, obesity prevalence was re-estimated after excluding participants with prevalent CVD at baseline to better align with a primary prevention setting. Kaplan-Meier survival curves were used to describe the cumulative incidence of total CVD, CVD mortality, and its subtypes over follow-up, separately for men and women.

For the longitudinal analysis, the associations of the new definition of obesity, as well as obesity phenotypes, with incident total CVD, CVD mortality, and its subtypes were assessed separately in men and women using Cox proportional hazards models with age as the time scale. Hazard ratios (HRs) and 95% confidence intervals (CIs) were estimated after adjustment for education level, smoking status, and family history of CVD (Model 1). Model 2 further adjusted for CKD, diabetes, hypertension, HDL-C, LDL-C, triglycerides, and lipid-lowering medication use to explore potential biological pathways underlying the observed associations. The proportional hazards assumption was evaluated using Schoenfeld's global test of residuals and was found to be satisfied. *P* values for interaction were calculated by including multiplicative interaction terms between obesity definition and sex for incident outcomes. Covariates were selected a *priori* based on clinical knowledge [[26], [27], [28]]. Potential multicollinearity among covariates was formally assessed using the variance inflation factor, and all variables included in the models had values below 5, indicating no significant multicollinearity.

In addition, we performed analyses modeling obesity as a time-varying covariate to account for changes in obesity status after the baseline TLGS visit, also including current smoking, CKD, diabetes, hypertension, and dyslipidemia as time-varying covariates to account for potential changes in these variables after the baseline visit but before the occurrence of events. In creating the time-varying covariates, measurements from TLGS phases 1, 2, 3 (2006–2008), 4 (2009–2011), 5 (2012–2015), 6 (2015–2018), and 7 (2018–2022) were incorporated into the regression analyses. Hypertension, diabetes, CKD, and dyslipidemia were considered to be present after onset [29]. When data were missing at a phase, we carried forward the most recent available value from an earlier examination until a new measurement was available. Across the seven phases, participants contributed a median of 5 to 6 attended examinations, with a mean of 4.80 (SD 2.04) to 4.96 (2.01) observed measurements per time-varying variable.

In secondary analyses, we [1] repeated the main analyses using the appropriate cutoff for elevated WC in the Iranian population [≥95 cm in both sexes [23]]; [2] examined the associations separately for CVD subtypes (i.e., CHD, MI, and stroke); [3] examined the associations with a composite of non-fatal MI and non-fatal stroke; [4] repeated the analyses for CVD mortality after excluding events occurring within the first 5 years of follow-up to reduce the possibility of reverse causality; [5] re-estimated the fully adjusted model using LDL-C derived from the modified Friedewald formula in place of the Martin/Hopkins estimate [30]; and [6] repeated the primary analyses using the traditional BMI-based definition of obesity.

As a sensitivity analysis, the nonparametric cumulative incidence function, implemented using the *cuminc* function in the *cmprsk* R package, was used to estimate the cumulative incidence of total CVD and CVD mortality while accounting for non-CVD death as a competing risk [31], and differences across obesity groups were assessed using Gray’s test. In addition, we re-estimated the associations of the new obesity definition and its phenotypes with total CVD and CVD mortality using *Fine & Gray* sub-distribution hazard models, with non-CVD mortality treated as the competing event [32]. All *P* values are two-sided, and *P* values <0.05 are considered statistically significant.

## 4 Results

At baseline, among 9429 individuals (mean age 47.7 years; 54.4% women), the prevalence of obesity was 26.3% under the traditional BMI-based definition. In contrast, the new *Lancet Commission* definition classified 67.2% as having obesity, including 26.2% with BMI-plus-anthropometric obesity and 41.0% with anthropometric-only obesity (Table S2 and Fig. 1). This pattern was observed in both men and women, although the relative contributions of obesity phenotypes differed by sex. Among participants with obesity under the traditional BMI-based definition, all men and 99.7% of women also met at least one anthropometric obesity criterion (WC, WHR, or WHtR), resulting in nearly complete overlap between the traditional and BMI-plus-anthropometric categories. Under the new definition, obesity prevalence increased markedly with age, rising from 53.7% in individuals aged <45 years to 81.7% in those aged ≥60 years. This age-related increase was largely driven by anthropometric-only obesity, which increased from 30.9% in the youngest group to 57.1% in the oldest. Similar findings were observed when applying Iranian WC cutoffs, with overall obesity prevalence reaching 65.1% and showing a comparable age gradient (50.9% to 81.0%). In sex-stratified analyses, the same pattern persisted; however, the contribution of anthropometric-only obesity to the overall prevalence was consistently more pronounced in men than in women across all age groups (Table S2).

**Fig. 1:**
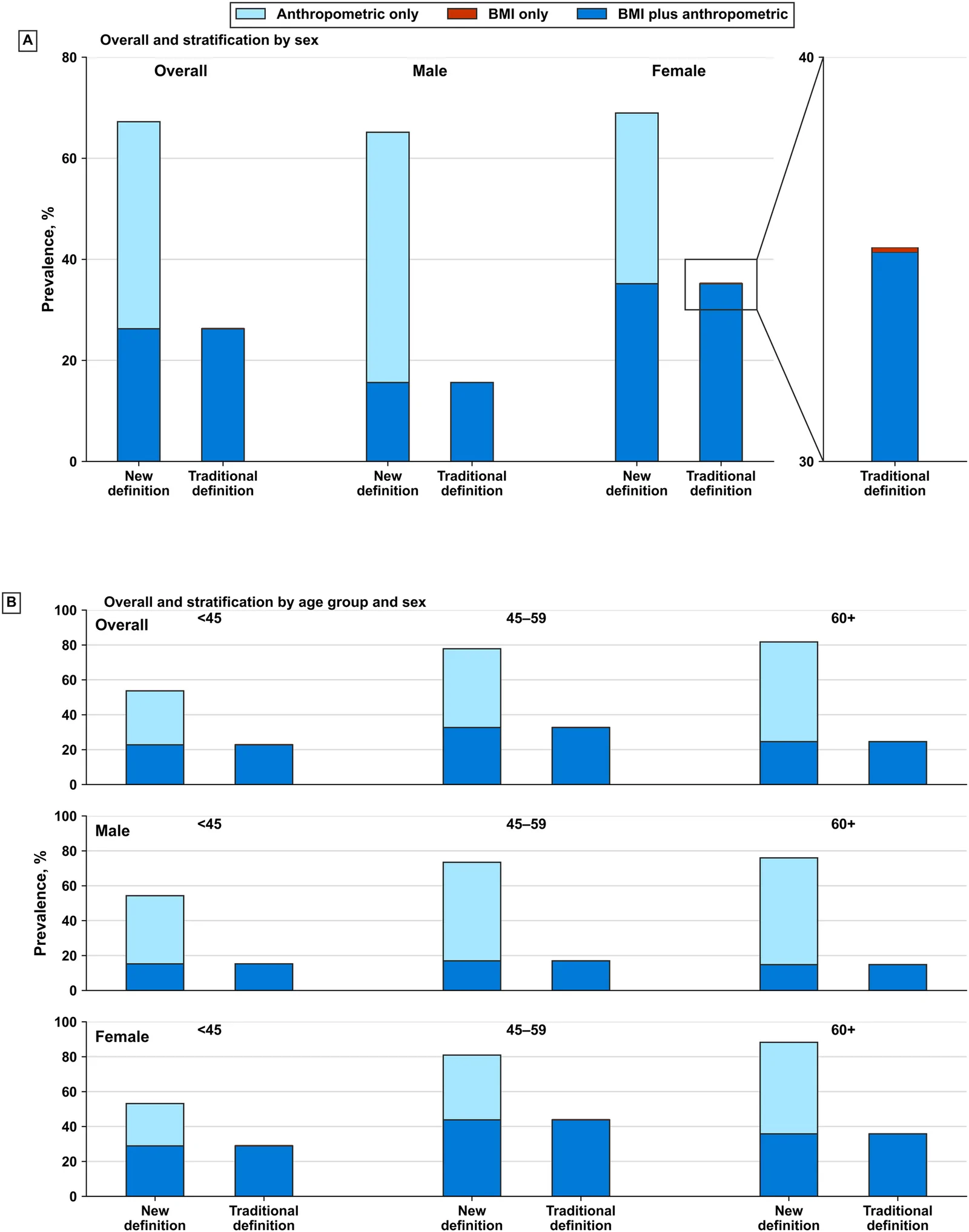
Prevalence of obesity by the traditional and new definitions in the TLGS cohort (n = 9429). Panel A shows overall obesity prevalence and obesity prevalence stratified by sex, while Panel B shows obesity prevalence across age groups overall and separately in men and women. The inset highlights the low prevalence of elevated BMI in the absence of elevated anthropometric measures under the traditional BMI-based obesity definition, shown in red in the female bar.

Among the 8835 participants free of prevalent CVD at baseline, the prevalence estimates were essentially comparable to those in the full cross-sectional sample, and a consistent pattern was observed across sex and age groups (Table S3).

For the longitudinal analysis, a total of 7781 participants (55.1% women) with a mean age of 46.8 (SD 12.3) years were included. Using the *Lancet Commission* definition, 3100 individuals (39.8%) were classified as having anthropometric-only obesity, 2042 (26.2%) as having BMI-plus-anthropometric obesity, and 2639 (34.0%) as being obesity-free.

The baseline characteristics of participants across obesity groups are presented in Table 1. Compared with the obesity-free group, individuals with any obesity phenotype were older and had a worse cardio-renal-metabolic profile, characterized by higher blood pressure, FPG, TC, LDL-C, and TG, along with lower HDL-C and eGFR levels (all *P* < 0.001). The BMI-plus-anthropometric obesity group had the highest mean BMI (33.3 vs. 23.8 kg/m^2^ in the obesity-free group), WC (102.3 vs. 78.7 cm), and WHtR (0.645 vs. 0.483). The anthropometric-only obesity group, despite having a mean BMI in the overweight range (26.7 kg/m²), exhibited a substantial burden of cardiometabolic risk factors, although generally at lower levels than those observed in the BMI-plus-anthropometric group. An exception was current smoking, which was more prevalent in the anthropometric-only group than in the BMI-plus-anthropometric group (18.1%vs. 10.5%). A higher proportion of women were in the BMI-plus-anthropometric group (72.9%), whereas men were more represented in the anthropometric-only group (54.9%) (all *P* < 0.05).

**Table 1:** Baseline characteristics of the study participants overall and stratified by the obesity phenotypes.

Characteristics	Total *(n=7781)*	Obesity-Free *(n=2639)*	With Obesity *(n=5142)*		*P* value
			Anthropometric only *(n=3100)*	BMI plus anthropometric *(n=2042)*	
Continuous variables, mean (SD)					
Age, years	46.8 (12.3)	42.2 (11.3)	50.2 (12.5)	47.7 (11.1)*	<0.001
BMI, kg/m²	27.5 (4.6)	23.8 (2.9)	26.7 (2.1)	33.3 (3.1)*	<0.001
WC, cm	90.6 (11.6)	78.7 (6.4)	93.0 (5.8)	102.3 (8.8)*	<0.001
WHR	0.893 (0.086)	0.820 (0.063)	0.940 (0.060)	0.916 (0.081)*	<0.001
WHtR	0.561 (0.078)	0.483 (0.041)	0.572 (0.040)	0.645 (0.057)*	<0.001
SBP, mmHg	120.9 (19.4)	113.1 (15.8)	123.8 (19.8)	126.6 (20.0)*	<0.001
DBP, mmHg	78.5 (10.9)	74.4 (9.7)	79.2 (10.7)	82.7 (10.8)*	<0.001
FPG, mg/dL	100.1 (34.3)	91.5 (21.4)	104.2 (39.5)	105.1 (37.2)	<0.001
eGFR, mL/min/1.73 m²	80.7 (14.1)	84.5 (13.8)	79.0 (14.0)	78.5 (13.7)	<0.001
TC, mg/dL	212.6 (45.4)	198.5 (41.5)	217.1 (45.3)	223.8 (45.8)*	<0.001
HDL-C, mg/dL	41.5 (11.0)	43.4 (11.4)	39.8 (10.5)	41.3 (10.7)*	<0.001
LDL-C, mg/dL	139.8 (37.8)	129.6 (35.7)	143.4 (37.7)	147.6 (38.0)*	<0.001
Triglycerides, median [IQR], mg/dL	155 [106–220]	116 [84–168]	170 [122–244]	182 [131–242]*	<0.001
Categorical variables, frequency (%)					
Women	4288 (55.1)	1401 (53.1)	1399 (45.1)	1488 (72.9)*	<0.001
Education					
<6	3100 (39.8)	642 (24.3)	1409 (45.5)	1049 (51.4)*	<0.001
6–12	3696 (47.5)	1513 (57.3)	1336 (43.1)	847 (41.5)*	
>12	985 (12.7)	484 (18.3)	355 (11.5)	146 (7.2)*	
Smoking status					
Never	5824 (74.9)	1930 (73.1)	2192 (70.7)	1702 (83.4)*	<0.001
Former	647 (8.3)	174 (6.6)	347 (11.2)	126 (6.2)*	
Current	1310 (16.8)	535 (20.3)	561 (18.1)	214 (10.5)*	
Family history of CVD	765 (9.8)	222 (8.4)	275 (8.9)	268 (13.1)*	<0.001
CKD	500 (6.4)	80 (3.0)	251 (8.1)	169 (8.3)	<0.001
Diabetes	745 (9.6)	82 (3.1)	385 (12.4)	278 (13.6)	<0.001
Antidiabetic medication use	353 (4.5)	44 (1.7)	187 (6.0)	122 (6.0)	<0.001
Hypertension	1887 (24.3)	273 (10.3)	865 (27.9)	749 (36.7)*	<0.001
Antihypertensive medication use	594 (7.6)	66 (2.5)	275 (8.9)	253 (12.4)*	<0.001
Lipid-lowering medication use	277 (3.6)	26 (1.0)	143 (4.6)	108 (5.3)	<0.001

Values are mean (standard deviation: SD) or median [interquartile range: IQR] for continuous variables and frequency (%) for categorical variables.

Abbreviations: BMI, body mass index; WC, waist circumference; WHR, waist-hip ratio; WHtR, waist-height ratio; SBP, systolic blood pressure; DBP, diastolic blood pressure; FPG, fasting plasma glucose; eGFR, estimated glomerular filtration rate; TC, total cholesterol; HDL-C, high-density lipoprotein cholesterol; LDL-C, low-density lipoprotein cholesterol; CVD, cardiovascular disease; CKD, chronic kidney disease.

⁎ *P* value <0.05 for comparison between individuals with obesity.

The cumulative incidence of CVD and cardiovascular mortality across obesity phenotypes, stratified by sex, is shown in Fig. 2. The number of individuals at risk to allow for better interpretation of the data is shown in Figures S2-S3. Over a median follow-up of 19.9 years (IQR, 15.8–20.5), 1450 participants developed CVD, 344 died from CVD, and 1254, 246, and 290 developed CHD, MI, and stroke, respectively. Crude event rates for both incident CVD and CVD mortality were higher in men than in women. In both sexes, the two obesity phenotypes were clearly separated from the obesity-free group for both endpoints, with significant overall differences across obesity-status groups (all log-rank *P* < 0.05). However, within each sex, no meaningful differences were observed between the anthropometric-only and BMI-plus-anthropometric groups (*P* > 0.05). Competing-risk cumulative incidence analyses, with non-CVD mortality treated as a competing event, yielded findings consistent with the primary Kaplan-Meier analyses, with significant differences across obesity-status groups using Gray’s test within each sex (all *P* < 0.05; Figures S4 and S5). The cumulative incidence curves for CVD subtypes are shown in Figure S6.

**Fig. 2:**
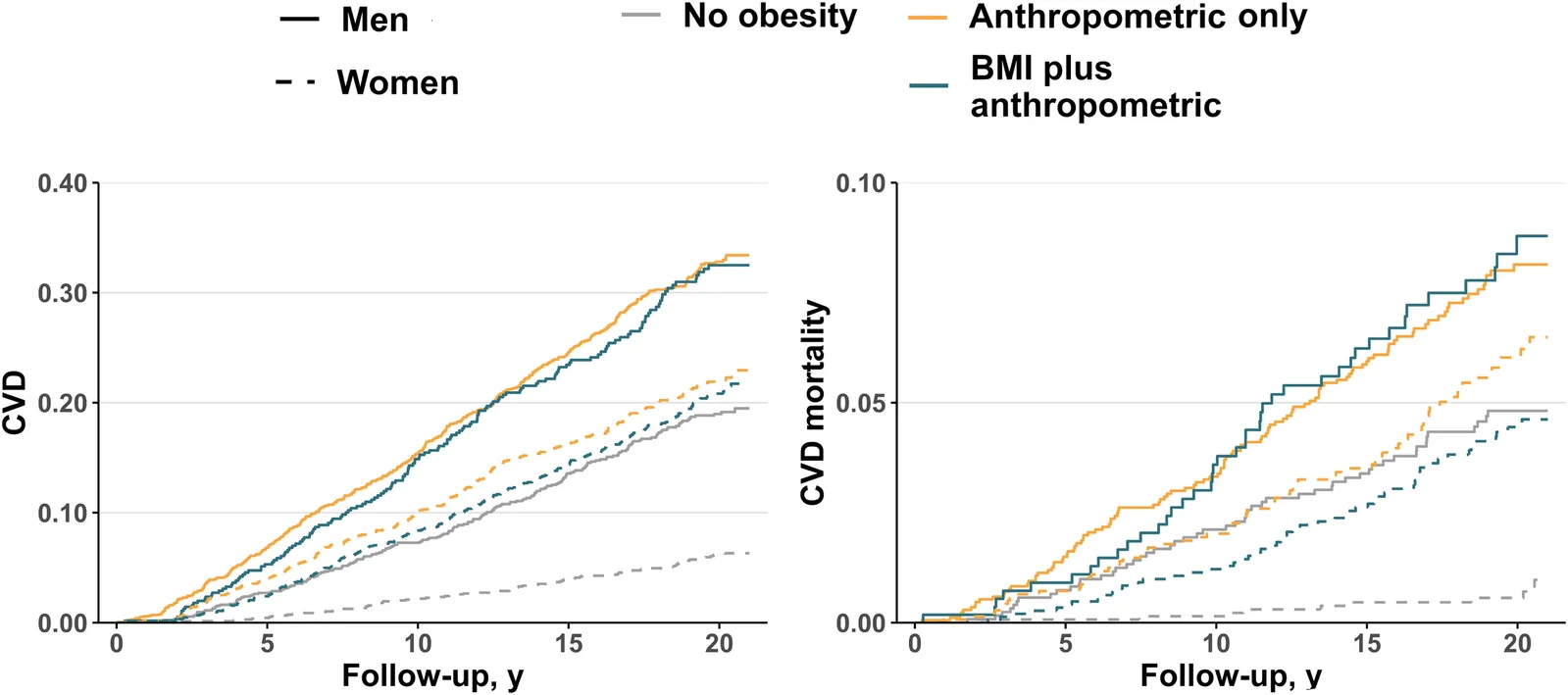
Cumulative incidence of CVD and CVD mortality across obesity phenotypes, stratified by sex. Solid lines indicate men; dashed lines indicate women. Abbreviations: CVD, cardiovascular diseases; BMI, body mass index.

The results from the multivariable Cox proportional hazards models, stratified by sex, are presented in Table 2 and Table S4. In pooled models including sex-obesity interaction terms, the new obesity definition showed significant interactions with sex for total CVD (*P_interaction_* = 0.005), CVD mortality (*P* = 0.01), and CHD (*P* = 0.02), but not for MI (*P* = 0.14) or stroke (*P* = 0.17).

**Table 2:** Association of the new definition of obesity with CVD and CVD mortality stratified by sex.

	Men					Women				
	Case/total	Model 1		Model 2		Case/total	Model 1		Model 2	
		HR (95% CI)	*P* value	HR (95% CI)	*P* value		HR (95% CI)	*P* value	HR (95% CI)	*P* value
CVD										
- No obesity	206/1238	1.00 (Reference)	…	1.00 (Reference)	…	75/1401	1.00 (Reference)	…	1.00 (Reference)	…
- With obesity	635/2255	1.48 (1.26–1.74)	<0.001	1.16 (0.98–1.37)	0.08	534/2887	1.91 (1.49–2.44)	<0.001	1.49 (1.16–1.92)	0.002
CVD mortality										
- No obesity	52/1238	1.00 (Reference)	…	1.00 (Reference)	…	9/1401	1.00 (Reference)	…	1.00 (Reference)	…
- With obesity	157/2255	1.26 (0.92–1.74)	0.15	1.08 (0.76–1.52)	0.67	126/2887	2.98 (1.49–5.92)	0.002	2.33 (1.16–4.71)	0.02

The interaction between obesity status and sex was statistically significant for both CVD and CVD mortality. For CVD, the P values for interaction were 0.005 in Model 1 and 0.020 in Model 2. For CVD mortality, the corresponding P values were 0.010 and 0.020, respectively.

Bold values indicate statistical significance (*P* < 0.05).

Hazard ratios (HRs) and 95% confidence intervals (CIs) from Cox proportional hazards models with age as time scale are reported.

Model 1 was adjusted for education level, smoking status, and family history of CVD.

Model 2 was additionally adjusted for chronic kidney disease, diabetes, hypertension, HDL-C, LDL-C, triglycerides, and lipid-lowering medication use.

Abbreviation: CVD: cardiovascular disease; HR, hazard ratio; CI, confidence interval; HDL-C, high-density lipoprotein cholesterol; LDL-C, low-density lipoprotein cholesterol.

In men, obesity, according to the new definition, was associated with significantly higher risks of total CVD (HR, 1.48, 95% CI, 1.26–1.74; *P* < 0.001), CHD (1.50, 1.27–1.78; *P* < 0.001), and MI (2.12, 1.43–3.13; *P* < 0.001) compared with no obesity. The association with stroke did not reach statistical significance (1.44, 0.98–2.10; *P* = 0.06), nor did the association with CVD mortality (1.26, 0.92–1.74; *P* = 0.15). After further adjustment in Model 2, the association with MI remained statistically significant (1.55, 1.03–2.32; *P* = 0.03), whereas the associations with total CVD and CHD were attenuated to 16% and 17% higher risks, respectively, and were no longer statistically significant (both *P* = 0.08) (Table 2 and Table S4).

Women with obesity had a significantly higher risk of total CVD (HR 1.91, 95% CI 1.49–2.44; *P* < 0.001), CVD mortality (2.98, 1.49–5.92; *P* = 0.002), CHD (1.81, 1.39–2.36; *P* < 0.001), MI (3.06, 1.31–7.15; *P* = 0.01), and stroke (2.17, 1.16–4.06; *P* = 0.02) compared with obesity-free women in Model 1. The associations for total CVD, CVD mortality, CHD, and stroke remained statistically significant in Model 2. For MI, although obesity was associated with a 2.16-fold higher risk, the association did not reach statistical significance (2.16, 0.91–5.13; *P* = 0.08) (Table 2 and Table S4).

The results of analyses examining the association of the two components of the new obesity definition, "anthropometric-only" and "BMI-plus-anthropometric" obesity, with CVD and CVD mortality are shown in Fig. 3. For men, Model 1 showed significant associations between CVD risk and both the anthropometric-only phenotype (HR 1.45, 95% CI 1.23–1.71; *P* < 0.001) and the BMI-plus-anthropometric phenotype (1.62, 1.31–2.00; *P* < 0.001). However, in Model 2, these associations attenuated and were no longer statistically significant (*P* = 0.08 and *P* = 0.26, respectively). For CVD mortality, only the BMI-plus-anthropometric phenotype was significantly associated with risk in Model 1 (1.61, 1.06–2.45; *P* = 0.03), while the anthropometric-only phenotype was not statistically significant (1.18, 0.85–1.64; *P* = 0.34).

**Fig. 3:**
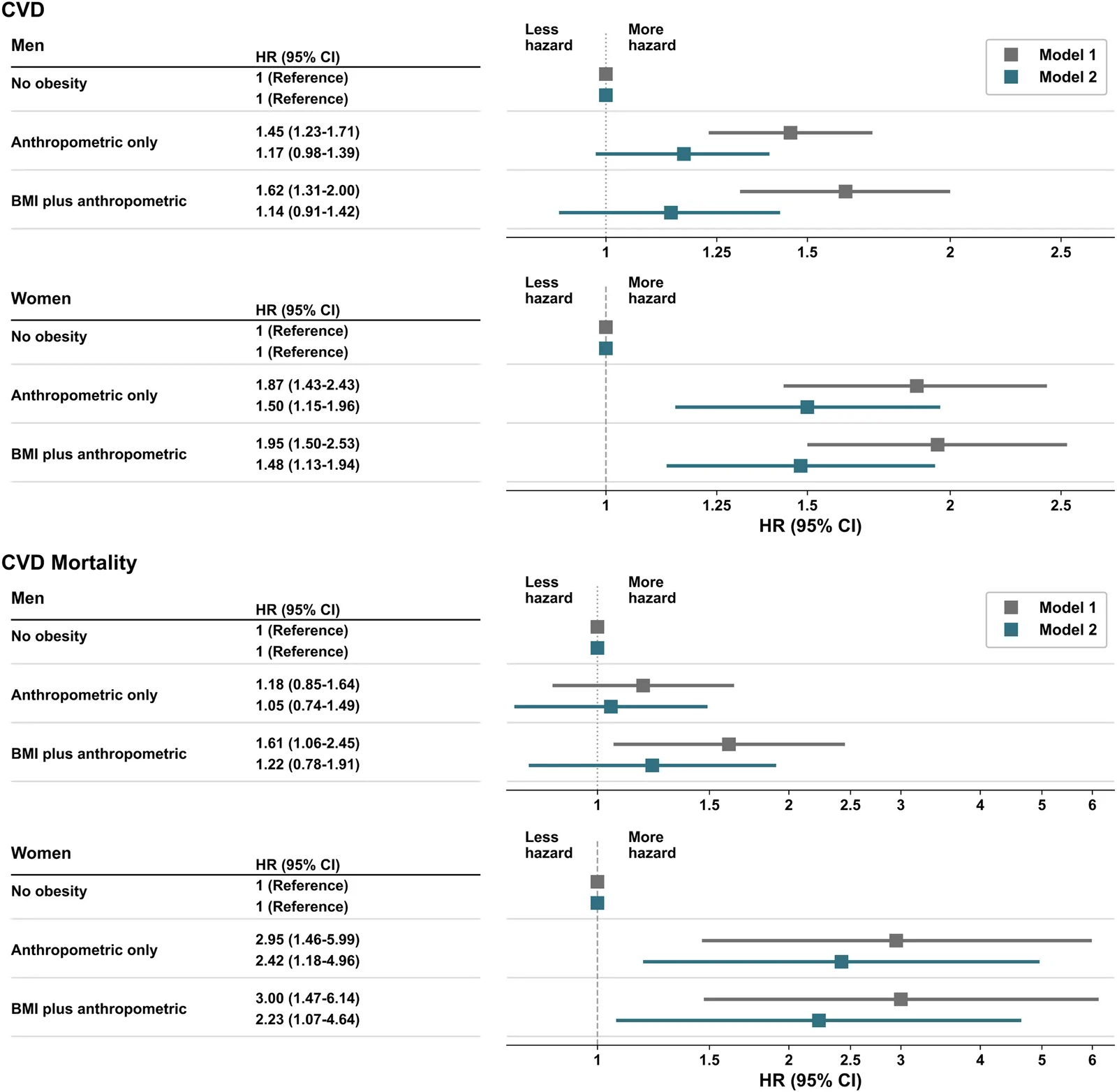
Association of obesity phenotypes with CVD and CVD mortality, stratified by sex. Hazard ratios (HRs) and 95% confidence intervals (CIs) from Cox proportional hazards models with age as time scale are reported. Model 1 was adjusted for education level, smoking status, and family history of CVD. Model 2 was additionally adjusted for chronic kidney disease, diabetes, hypertension, HDL-C, LDL-C, triglycerides, and lipid-lowering medication use. Abbreviations: CVD, cardiovascular diseases; HDL-C, high-density lipoprotein cholesterol; LDL-C, low-density lipoprotein cholesterol; BMI, body mass index.

For women, Model 1 showed significant associations of both the anthropometric-only and BMI-plus-anthropometric phenotypes with CVD (1.87 [95% CI 1.43–2.43; *P* < 0.001] and 1.95 [1.50–2.53; *P* < 0.001], respectively) and CVD mortality (2.95 [1.46–5.99; *P* = 0.003] and 3.00 [1.47–6.14; *P* = 0.003], respectively). In Model 2, the associations for women remained statistically significant for both CVD and CVD mortality (Fig. 3). Results for the CVD subtypes are shown in Figure S7, which were essentially in line with the higher risk observed for total CVD in Model 1.

The results using Iranian-specific WC cutoffs are presented in Table S5 and were largely consistent with the primary analyses, except that the association between obesity and MI in women became statistically significant in Model 2 (HR 2.29, 95% CI 1.02–5.13; *P* = 0.04). In a composite analysis of non-fatal MI and non-fatal stroke, results were consistent with the primary analyses based on total CVD for both sexes, with the new obesity definition and the BMI-plus-anthropometric phenotype remaining associated with the outcome after full adjustment in women, and the BMI-plus-anthropometric phenotype also remaining significant in men (Table S6). The main difference was that the interaction with sex was no longer statistically significant (*P* interaction = 0.17). To address potential reverse causality, early CVD mortality cases occurring within the first 5 years of follow-up were excluded. Findings consistent with the primary analyses for both sexes were observed (Table S7). When the fully adjusted model was re-estimated using LDL-C derived from the modified Friedewald formula instead of the Martin/Hopkins estimate, the results were consistent with the primary analyses (Table S8). Use of the conventional BMI-only obesity definition in place of the new definition produced more attenuated associations with CVD outcomes (Table S9). In Model 1, BMI-only obesity was associated with incident CVD in men (HR 1.26, 95% CI 1.06–1.51; *P* = 0.009) and women (1.24, 1.05–1.46; *P* = 0.009), and with CVD mortality in men (1.44, 1.02–2.04; *P* = 0.039); however, none of these associations remained statistically significant after full adjustment in Model 2.

In competing-risk analyses treating non-CVD mortality as a competing event, sub-distribution HRs were nearly identical with the estimates from the primary Cox models (Table 3). In Model 1, obesity according to the new definition was associated with incident total CVD in both men (sub-hazard ratio [sHR] 1.50, 95% CI 1.28–1.76; *P* < 0.001) and women (1.91, 1.49–2.46; *P* < 0.001). For CVD mortality, the association was evident in women (2.97, 1.50–5.90; *P* = 0.002), but not in men (1.25, 0.91–1.72; *P* = 0.17). After full adjustment, the stronger associations among women persisted, and the results for both obesity phenotypes remained broadly consistent with the primary analyses. Notably, among men, the associations of the new obesity definition (1.18, 1.00–1.40; *P* = 0.049) and the anthropometric-only obesity phenotype (1.19, 1.01–1.42; *P* = 0.043) with incident total CVD reached nominal statistical significance in the fully-adjusted models.

**Table 3:** Sex-stratified associations of the new obesity definition with CVD and CVD mortality, accounting for non-CVD mortality as a competing event.

	Men				Women			
	Model 1		Model 2		Model 1		Model 2	
	sHR (95% CI)	*P* value	sHR (95% CI)	*P* value	sHR (95% CI)	*P* value	sHR (95% CI)	*P* value
CVD								
							
- No obesity	1.00 (Reference)	…	1.00 (Reference)	…	1.00 (Reference)	…	1.00 (Reference)	…
- With obesity	1.50 (1.28–1.76)	<0.001	1.18 (1.00–1.40)	0.049	1.91 (1.49–2.46)	<0.001	1.50 (1.16–1.95)	0.002
- No obesity	1.00 (Reference)	…	1.00 (Reference)	…	1.00 (Reference)	…	1.00 (Reference)	…
- Anthropometric-only obesity	1.47 (1.25–1.74)	<0.001	1.19 (1.01–1.42)	0.043	1.90 (1.46–2.48)	<0.001	1.53 (1.17–2.02)	0.002
- BMI-plus-anthropometric obesity	1.60 (1.30–1.98)	<0.001	1.14 (0.91–1.43)	0.24	1.92 (1.48–2.51)	<0.001	1.48 (1.12–1.93)	0.005
CVD mortality								
							
- No obesity	1.00 (Reference)	…	1.00 (Reference)	…	1.00 (Reference)	…	1.00 (Reference)	…
- With obesity	1.25 (0.91–1.72)	0.17	1.12 (0.79–1.59)	0.52	2.97 (1.50–5.90)	0.002	2.36 (1.19–4.69)	0.01
- No obesity	1.00 (Reference)	…	1.00 (Reference)	…	1.00 (Reference)	…	1.00 (Reference)	…
- Anthropometric-only obesity	1.18 (0.84–1.65)	0.32	1.10 (0.74–1.56)	0.62	3.08 (1.52–6.21)	0.002	2.55 (1.27–5.14)	0.01
- BMI-plus-anthropometric obesity	1.49 (0.98–2.27)	0.06	1.23 (0.78–1.93)	0.37	2.84 (1.40–5.81)	0.004	2.15 (1.05–4.40)	0.04

Bold values indicate statistical significance (*P* < 0.05).

Sub-distribution hazard ratios and 95% confidence intervals were estimated using Fine and Gray competing-risk models, with non-CVD mortality treated as a competing event. For overall CVD, the event of interest was first fatal or non-fatal CVD, and non-CVD deaths occurring before any CVD event were treated as competing events (n = 457). For CVD mortality, CVD death was the event of interest, and non-CVD deaths were treated as competing events (n = 608).

Model 1 was adjusted for age, education level, smoking status, and family history of CVD.

Model 2 was additionally adjusted for chronic kidney disease, diabetes, hypertension, HDL-C, LDL-C, triglycerides, and lipid-lowering medication use.

Abbreviation: CVD: cardiovascular disease; sHR, sub-hazard ratio; CI, confidence interval.

The results of the time-varying Cox proportional hazards analyses examining the associations of the new obesity definition and its phenotypes with incident total CVD, CVD mortality, and its subtypes are presented in Table 4 and Table S10-S11).

**Table 4:** Sex-stratified associations of the new definition of obesity, modeled as a time-varying covariate, with CVD and CVD mortality.

	Men				Women			
	Model 1		Model 2		Model 1		Model 2	
	HR (95% CI)	*P* value	HR (95% CI)	*P* value	HR (95% CI)	*P* value	HR (95% CI)	*P* value
CVD								
- No obesity	1.00 (Reference)	…	1.00 (Reference)	…	1.00 (Reference)	…	1.00 (Reference)	…
- With obesity	1.50 (1.21–1.85)	<0.001	1.24 (1.00–1.54)	0.051	2.64 (1.70–4.08)	<0.001	2.06 (1.32–3.21)	0.001
- No obesity	1.00 (Reference)	…	1.00 (Reference)	…	1.00 (Reference)	…	1.00 (Reference)	…
- Anthropometric-only obesity	1.45 (1.16–1.80)	<0.001	1.24 (1.00–1.55)	0.053	2.46 (1.57–3.84)	<0.001	2.05 (1.30–3.22)	0.002
- BMI-plus-anthropometric obesity	1.67 (1.31–2.14)	<0.001	1.23 (0.95–1.59)	0.109	2.80 (1.80–4.36)	<0.001	2.08 (1.32–3.26)	0.001
CVD mortality								
- No obesity	1.00 (Reference)	…	1.00 (Reference)	…	1.00 (Reference)	…	1.00 (Reference)	…
- With obesity	0.96 (0.68–1.34)	0.80	0.85 (0.60–1.21)	0.37	1.04 (0.53–2.06)	0.90	0.82 (0.41–1.64)	0.58
- No obesity	1.00 (Reference)	…	1.00 (Reference)	…	1.00 (Reference)	…	1.00 (Reference)	…
- Anthropometric-only obesity	0.89 (0.63–1.26)	0.52	0.82 (0.57–1.18)	0.28	1.01 (0.51–2.03)	0.97	0.85 (0.42–1.70)	0.64
- BMI-plus-anthropometric obesity	1.22 (0.81–1.85)	0.34	0.99 (0.64–1.53)	0.98	1.08 (0.53–2.19)	0.83	0.80 (0.39–1.63)	0.53

Bold values indicate statistical significance (P < 0.05).

Hazard ratios and 95% confidence intervals were estimated using time-varying Cox proportional hazards models, in which both the primary exposure and covariates were modeled as time-dependent variables (except for education level and family history of CVD).

Model 1 was adjusted for age, education level, smoking status, and family history of CVD.

Model 2 was additionally adjusted for chronic kidney disease, diabetes, hypertension, and dyslipidemia.

Abbreviation: CVD: cardiovascular disease; HR, hazard ratio; CI, confidence interval.

Analogous findings for men regarding the association between obesity and total CVD were observed when obesity and current smoking were modeled as time-varying covariates in Model 1 to account for changes in obesity and current smoking after the baseline TLGS visit. In women, the association was even more pronounced than that observed in the baseline analysis, with obesity associated with a 2.64-fold higher risk of total CVD (95% CI 1.70–4.08; *P* < 0.001). A similar pattern was observed for obesity phenotypes in both sexes. For CVD mortality, however, the associations were attenuated to the null in women. When CKD, diabetes, hypertension, and dyslipidemia from TLGS visits 1 through 7 were further modeled as time-varying covariates in Model 2, the adjusted HRs for incident total CVD associated with obesity were 1.24 (1.00–1.54; *P* = 0.051) in men and 2.06 (1.32–3.21; *P* = 0.001) in women (Table 4). For CVD subtypes, obesity defined by the new definition was no longer statistically significantly associated with MI in women. However, the association between obesity and CHD became statistically significant for men in model 2 (HR 1.28; 95% CI 1.01–1.62; *P* = 0.03). The remaining associations were consistent with the primary analyses (Table S10).

## 5 Discussion

In this analysis of the TLGS cohort, applying the *Lancet Commission's* new definition increased obesity prevalence from 26.3% to 67.2%. Nearly all participants meeting the traditional BMI-based definition also met at least one anthropometric criterion, and similar results were observed using Iranian-specific WC cutoffs. Prevalence increased markedly with age, from 53.7% in those aged <45 years to 81.7% in those aged ≥60 years. Although prevalence was similar between sexes (65.1% in men vs. 69.0% in women), its cardiovascular impact was greater in women. The new obesity definition was associated with a 48% higher risk of CVD in men and with 91% and three-fold higher risks of total CVD and CVD mortality in women, respectively. It was also associated with higher risks of CVD subtypes in both sexes, ranging from 44% to 112% in men and from 81% to 206% in women. For total CVD and CVD mortality, after further adjustment for potential mediators, associations were attenuated in men, whereas in women the associations remained statistically significant. Importantly, when non-CVD death was treated as a competing event, the fully adjusted associations of both the new obesity definition and the anthropometric-only phenotype with incident CVD reached nominal statistical significance in men.

In our TLGS cohort, 65.1% of men and 69.0% of women met criteria for obesity under the new definition, compared with 15.6% and 35.3%, respectively, under the traditional BMI-based definition. This higher prevalence was explained entirely by the inclusion of anthropometric-only obesity. Additionally, in nearly all individuals with BMI-defined obesity, obesity was also supported by at least one additional anthropometric measure, consistent with findings from previous studies in U.S. and European populations [22,33]. Previously, Stein et al. [20] assessed the prevalence of the new obesity definition among 290,664 UK Biobank participants and reported that 36% had preclinical obesity and 15% had clinical obesity (excess body fat with organ dysfunction). In the All of Us (AoS) cohort, 68.6% of participants met the *Lancet Commission* criteria for obesity, corresponding to a 60% increase over the conventional BMI-based definition [22]. Another study based on a representative U.S. population reported that use of the “*Excess Adiposity*” criteria, which also incorporated dual-energy X-ray absorptiometry (DXA) body fat percentage, increased the prevalence of obesity to >60% of U.S. adults; furthermore, ∼45 million adults with a normal or overweight BMI were newly classified as having obesity under the “*Excess Adiposity*” criteria [34].

Of note, nearly 80% of participants aged 45 to 59 years and >80% of those aged 60 years or older in the TLGS cohort met criteria for obesity. The greater prevalence of anthropometric-only obesity in older adults is in line with previous studies showing that, with advancing age and particularly around menopause, central fat accumulation becomes more pronounced [22,[35], [36], [37]].

Our analyses suggest that the new definition of obesity identifies individuals at higher long-term risk of CVD, CVD mortality, and its subtypes, although the strength and persistence of these associations differed by sex and by the degree of adjustment. After adjustment for sociodemographic and behavioral factors, the new definition was associated with a 48% higher risk of CVD in men and a 91% higher risk in women, an approximately 3-fold higher risk of CVD mortality in women, and 44% to 112% higher risks of CHD, MI, and stroke in men versus 81% to 206% in women. After further adjustment for cardiometabolic risk factors, which lie on the causal pathway linking obesity to CVD, the associations in men with total CVD and CHD were attenuated to non-significance, and only MI remained significant, suggesting that much of the excess risk in men is accounted for by these mediators. In women, by contrast, the associations with CVD, CVD mortality, CHD, and stroke remained significant after full adjustment. Of note, when non-CVD death was treated as a competing event rather than as censoring, the fully adjusted association between the new obesity definition and incident CVD in men became statistically significant. In a European population-based cohort study of 2606 individuals, >80% of adults with confirmed obesity (BMI ≥30 kg/m^2^ incorporating ≥1 anthropometric measure) met criteria for clinical obesity, and this group had a 2.8-fold higher risk of incident CVD (defined as fatal or nonfatal MI and stroke) than adults without obesity not meeting clinical criteria [33]. However, preclinical obesity was not associated with an increased risk of cardiovascular disease [33]. It is important to note that the preclinical obesity comprised only 29 individuals. In another study, among UK Biobank participants, both preclinical and clinical obesity were associated with a graded increase in CVD mortality compared with no obesity, with risks higher by 38% and 126%, respectively [20].

Regarding obesity phenotypes, in women, both BMI-plus-anthropometric obesity and anthropometric-only obesity were associated with higher risks of CVD, CVD mortality, and its subtypes, with risks ranging from 1.79- to 3.00-fold and 1.84- to 3.45-fold, respectively, indicating comparable risk across the two phenotypes; the associations with CVD and CVD mortality, to lesser extent with CHD and stroke, remained significant after full adjustment for cardiometabolic risk factors. In men, only the association between anthropometric-only obesity and MI remained statistically significant after full adjustment for cardiometabolic risk factors. For total CVD, the fully adjusted association became nominally significant only in competing-risk analyses accounting for non-CVD death (sHR, 1.19; 95% CI, 1.01–1.42). Previously, among 301,026 individuals in the AoS cohort, longitudinal analyses showed that both BMI-plus-anthropometric obesity and anthropometric-only obesity were associated with higher risks of cardiovascular events (i.e., MI, stroke, and acute heart failure), with 81% and 55% higher risks, respectively [22]. BMI alone is not a perfect adiposity index because it does not adequately capture visceral fat, which is strongly associated with inflammation, insulin resistance, diabetes, CVD, and other adverse outcomes [38,39]. In this context, the high prevalence of anthropometric-only obesity among our participants supports the use of anthropometric measures alongside BMI to identify individuals with excess abdominal adiposity despite having a BMI < 30 kg/m^2^ [39,40]. This distinction is clinically relevant, as “*Normal-Weight Central Obesity*” is already shown to increase the risk of cardiometabolic and cardiovascular risk [39,[41], [42], [43]]. Given that anthropometric-only obesity and BMI-plus-anthropometric obesity may represent biologically and clinically distinct phenotypes, management strategies for anthropometric-only obesity call for further research. Given evidence that exercise can reduce visceral adiposity [[44], [45], [46], [47]], this phenotype may warrant more targeted investigation in future intervention studies.

Replacing the new definition with the conventional BMI-based definition produced weaker associations that did not persist after full adjustment. By incorporating central adiposity, the framework reclassifies a sizeable proportion of conventionally non-obese individuals as having obesity and identifies excess cardiovascular risk that the BMI-only definition did not capture, since the latter retains these higher-risk individuals within the no obesity reference group. These observations align with the All of Us cohort, in which anthropometric-only obesity was independently associated with incident cardiovascular events [22], and with earlier evidence that normal-weight central obesity carries elevated cardiovascular mortality [43].

In contrast to the findings from the UK Biobank cohort [20], our study revealed pronounced sex disparities in the associations of the new obesity definition with CVD, CVD mortality, and CHD, which persisted when WC cutoffs appropriate for the Iranian population were applied. The effect of obesity on cardiovascular endpoints was more pronounced in women, which is in line with the observation that women without obesity under the *Lancet Commission* framework had the lowest cumulative incidence of cardiovascular events. The associations in women remained significant after controlling for potential mediators, with obesity still associated with 1.37- to 2.33-fold higher risks of CVD, CVD mortality, and CHD. In time-updated analyses accounting for incident hypertension, CKD, diabetes, and dyslipidemia over follow-up, time-updated obesity remained strongly associated with CVD and CHD in women, although the association with CVD mortality was attenuated. Our findings are in line with a recent meta-analysis of 31 prospective studies, which showed that the association of CHD with both WC (relative risk, 1.80 vs 1.27 for highest vs. lowest categories) and WHR (1.89 vs 1.50) was stronger in women than in men [48]. The observed sex difference is also in line with results from two community-based cohort studies that showed a stronger relationship between central obesity measures and CVD in women than in men [49]. Results from previous studies among the European populations also suggested that central obesity measures were more strongly associated with CVD mortality than BMI, with some evidence that these differences were more pronounced in women than in men [50]. Also, in the UK Biobank, it was previously reported that higher WC and WHR conferred a greater excess risk of MI in women than in men, with women-to-men ratios of HRs of 1.07 and 1.15, respectively [51]. Previously, we have shown that there are some indications of stronger associations between metabolic phenotypes of obesity and incident cardio-renal-metabolic (CRM) multimorbidity among women than men [52]. However, a systematic review and meta-analysis of 95 cohorts with 1·2 million participants found no evidence of sex disparities in the association of increased BMI with the risk of CHD across diverse populations [53]. Thus, further research on sex disparities regarding the new obesity definition is warranted.

Notably, the interaction with sex was largely confined to total CVD, CVD mortality, and CHD, and was not apparent for the compsoite of non-fatal MI and stroke; although the fully adjusted associations were significant for MI in men and stroke in women, the non-significant interaction terms preclude inference of sex-specific susceptibility to either event. Accordingly, when non-fatal MI and non-fatal stroke were analyzed as a single composite excluding CVD death, the interaction with sex was no longer signifcant, and obesity was associated with a higher risk of the composite in Model 1 for both men and women, of a magnitude comparable to that observed for total CVD and CHD; after full adjustment, this association remained significant in women but, as with total CVD and CHD, was attenuated to non-significance in men, with only the BMI-plus-anthropometric phenotype remaining significant. This pattern suggests that the sex divergence in our cohort was driven largely by CVD mortality and non-hard CHD events rather than by non-fatal hard CVD events, consistent with reports that central adiposity is more strongly associated with cardiovascular mortality [28] in women than in men, and may reflect sex differences in the burden and prognostic weight of accompanying cardiometabolic risk factors [54]. We therefore repeated the analyses to assess the impact of non-CVD death as a competing event on the observed sex difference. This is particularly relevant where non-CVD mortality is common, such as among men and older adults [55,56]. In men, cause-specific and sub-distribution hazard models produced near-identical point estimates. In cause-specific models, the fully adjusted associations of the new obesity definition and anthropometric-only obesity with incident CVD were similar, each indicating an approximately 16% higher risk, but neither was statistically significant. After accounting for non-CVD death as a competing event, the corresponding fully adjusted estimates reached nominal significance for both the new definition (sHR, 1.18; 95% CI, 1.00–1.40) and anthropometric-only obesity (sHR, 1.19; 95% CI, 1.01–1.42), highlighting the importance of considering competing risk in the interpretation of our findings regarding men.

Several factors may explain the observed sex disparities. Although obesity affects both sexes, its cardiovascular consequences differ, in part because adipose tissue is distributed differently in men and women under the influence of sex hormones [54,57]. Abdominal fat accumulation, which is captured more directly by anthropometric measures than by BMI, can trigger systemic inflammation and is closely linked with cardiovascular risk [58]. Age-related changes in body fat distribution, including the shift toward central adiposity that accompanies the menopausal transition, may further contribute to the greater burden of central obesity in women [59]. In exploratory analyses stratified by age relative to the average age at menopause among Iranian women (48.2 years) [60], women younger and older than this age threshold showed a 73% and 97% significantly higher risk of incident CVD, respectively (data not shown); however, individual-level data on menopausal status and hormone therapy were unavailable, so these factors could not be formally assessed. The greater cardiovascular vulnerability observed in women may also reflect female-specific risk enhancers [61], such as adverse pregnancy outcomes, including hypertensive disorders of pregnancy, gestational diabetes, and preterm delivery, which are not uncommon among Iranian women [[62], [63], [64]].

**Figure:**
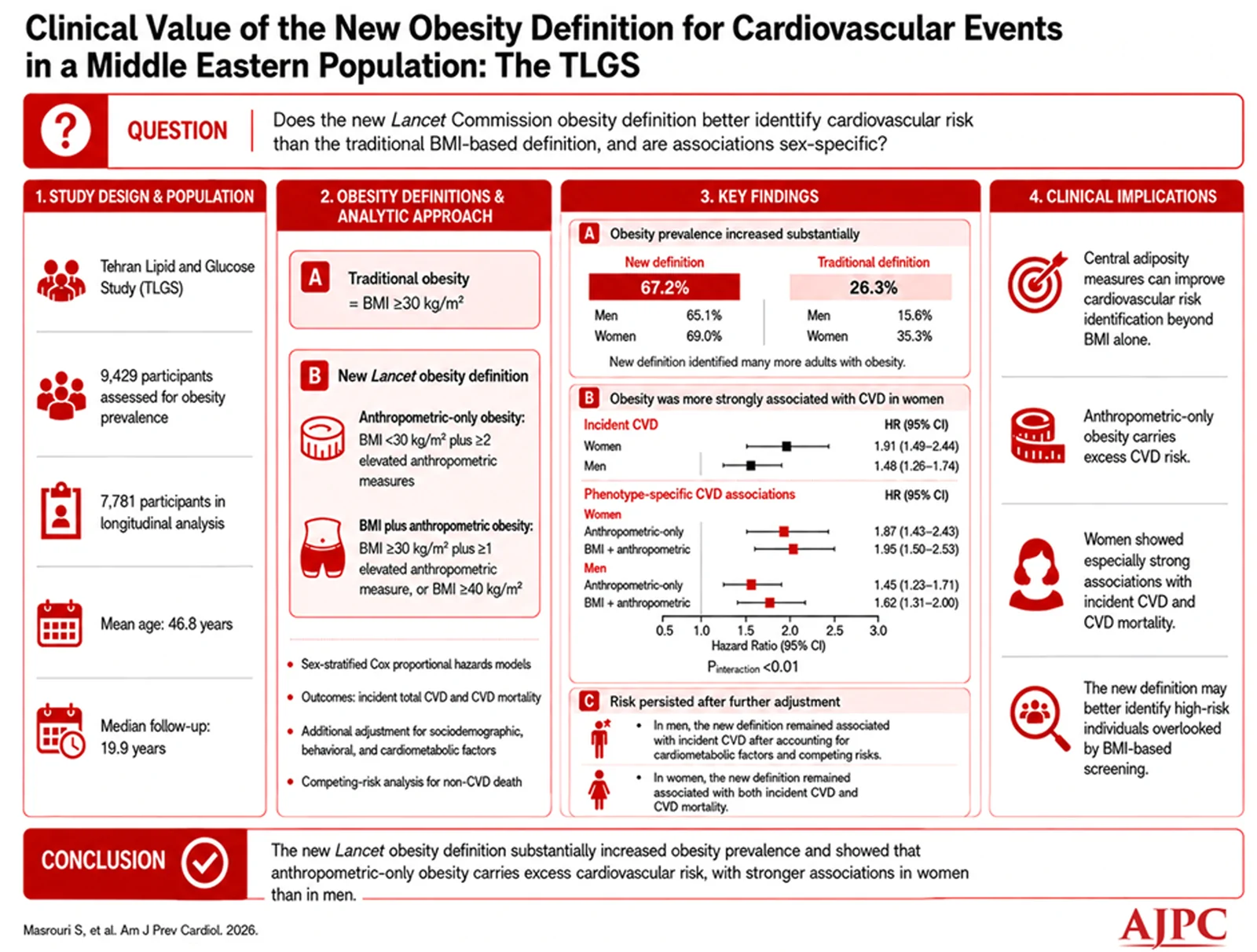
Central Illustration.

## 6 Limitations

Several limitations should be considered when interpreting our findings. First, as an observational study, residual confounding cannot be excluded despite adjustment for major cardiovascular risk factors; unmeasured factors such as inflammation, menopausal status, HRT use, diet, socioeconomic status, and healthcare access may have influenced the results. Second, because the TLGS includes urban residents of Tehran, the findings may not be generalizable to rural areas or to other racial/ethnic groups. Third, relatively few participants had class II or class III BMI-defined obesity, accounting for 4.7% and 1.1% of the cohort, respectively; therefore, the BMI-defined obesity burden in our study was driven largely by class I BMI-defined obesity. Fourth, some baseline data, including medical history and smoking status, were self-reported and therefore subject to misclassification. Fifth, DXA scans were not available in TLGS and could not be included in the assessment of central adiposity; if available, they would likely have increased rather than reduced the estimated prevalence of obesity. Sixth, central adiposity was defined using standard U.S. cutoffs for WHR and WHtR, although optimal risk-based thresholds may differ across populations. In sensitivity analyses, we used an appropriate cutoff point for elevated WC in the Iranian population, which yielded results consistent with the main analysis. Seventh, we were unable to assess clinical obesity because data on organ dysfunction and/or physical limitations were unavailable. Finally, in the absence of a gold standard for defining obesity, the value of the new framework was assessed indirectly through its ability to stratify future CVD risk.

## 7 Conclusions

Our study showed that applying the *Lancet Commission's* new obesity definition increased obesity prevalence to over 60%. The new definition and its phenotypes were associated with CVD events in both sexes after adjustment for sociodemographic and behavioral factors; with further adjustment for cardiometabolic factors lying on the causal pathway between obesity and CVD, these associations persisted in women and were largely attenuated in men, although a modest association in men reached nominal significance when the competing risk of non-CVD death was taken into account. These findings support the inclusion of anthropometric-only obesity within this framework, as it identifies individuals, particularly women, at higher risk of cardiovascular events. At the same time, the results highlight important knowledge gaps regarding clinical obesity and underscore the need for further research to inform evidence-based care in populations from the MENA region.

## Ethics approval and consent to participate

This study was approved by the Institutional Review Board of the Research Institute of Endocrine Sciences, Shahid Beheshti University of Medical Sciences, Tehran, Iran. The study was conducted in accordance with the principles of the Declaration of Helsinki. Written informed consent was obtained from all participants.

## Availability of data and materials

The datasets used and analyzed during the current study are available from the corresponding author upon reasonable request.

## Funding

A part of this work has been funded by the Iran National Science Foundation (INSF) project No. 40408192.

## CRediT authorship contribution statement

**Soroush Masrouri:** Writing – review & editing, Writing – original draft, Software, Methodology, Investigation, Formal analysis, Data curation, Conceptualization. **Parto Hadaegh:** Writing – review & editing, Writing – original draft. **Navid Ebrahimi:** Writing – review & editing, Writing – original draft, Methodology, Investigation. **Amir Abdi:** Writing – original draft. **Fereidoun Azizi:** Validation, Supervision, Project administration. **Farzad Hadaegh:** Writing – review & editing, Writing – original draft, Validation, Supervision, Project administration, Methodology, Investigation, Funding acquisition, Data curation, Conceptualization.

## Declaration of competing interest

The authors declare that they have no known competing financial interests or personal relationships that could have appeared to influence the work reported in this paper.
