# Supplementary material for: Clinical value of the new obesity definition for cardiovascular events in a Middle Eastern population: the TLGS
Source: Am J Prev Cardiol. 2026 Jul 23;30:101766. doi: 10.1016/j.ajpc.2026.101766 (PMC13631882; doi:10.1016/j.ajpc.2026.101766)
Supplement: Supplementary file 1 [file mmc1.pdf]

Supplementary Materials

Clinical value of the new obesity definition for cardiovascular events in a Middle Eastern population: the TLGS

Soroush Masrouri, MD; Parto Hadaegh, MS; Navid Ebrahimi, MD; Amir Abdi, MD; Fereidoun Azizi, MD; and Farzad Hadaegh, MD

Figure S1. Flowchart of the study population.

Figure S2. Kaplan-Meier cumulative incidence of CVD by obesity phenotype and sex

Figure S3. Kaplan-Meier cumulative incidence of CVD mortality by obesity phenotype and sex

Figure S4. Cumulative incidence function of CVD by obesity phenotype and sex, accounting for non-cardiovascular death as a competing event.

Figure S5. Cumulative incidence function of CVD mortality by obesity phenotype and sex, accounting for non-cardiovascular death as a competing event.

Figure S6. Cumulative incidence of cardiovascular event subtypes across obesity phenotypes, stratified by sex

Figure S7. Association of obesity phenotypes with CHD, MI, and stroke, stratified by sex

Table S1. Definitions of obesity

Table S2. Prevalence of obesity by the traditional and new definitions among the TLGS cohort participants

Table S3. Prevalence of obesity under the traditional and new definitions among participants free of prevalent cardiovascular disease at baseline

Table S4. Association of the new definition of obesity with cardiovascular event subtypes, stratified by sex

Table S5. Association of the new obesity definition with cardiovascular events using Iranian waist circumference cut-offs for elevated WC, stratified by sex

Table S6. Association of the new definition of obesity with a composite of nonfatal MI and nonfatal stroke stratified by sex

Table S7. Associations of obesity phenotypes with CVD mortality after excluding events occurring within the first 5 years of follow-up

Table S8. Association of the new definition of obesity with CVD and CVD mortality stratified by sex, with adjustment for LDL-C estimated using the modified Friedewald formula

Table S9. Association of the traditional definition of obesity with CVD and CVD mortality stratified by sex

Table S10. Sex-stratified associations of the new definition of obesity, modeled as a time-varying covariate, with cardiovascular event subty

Table S11. Availability of variables included in the time-varying analyses across seven study visitspes

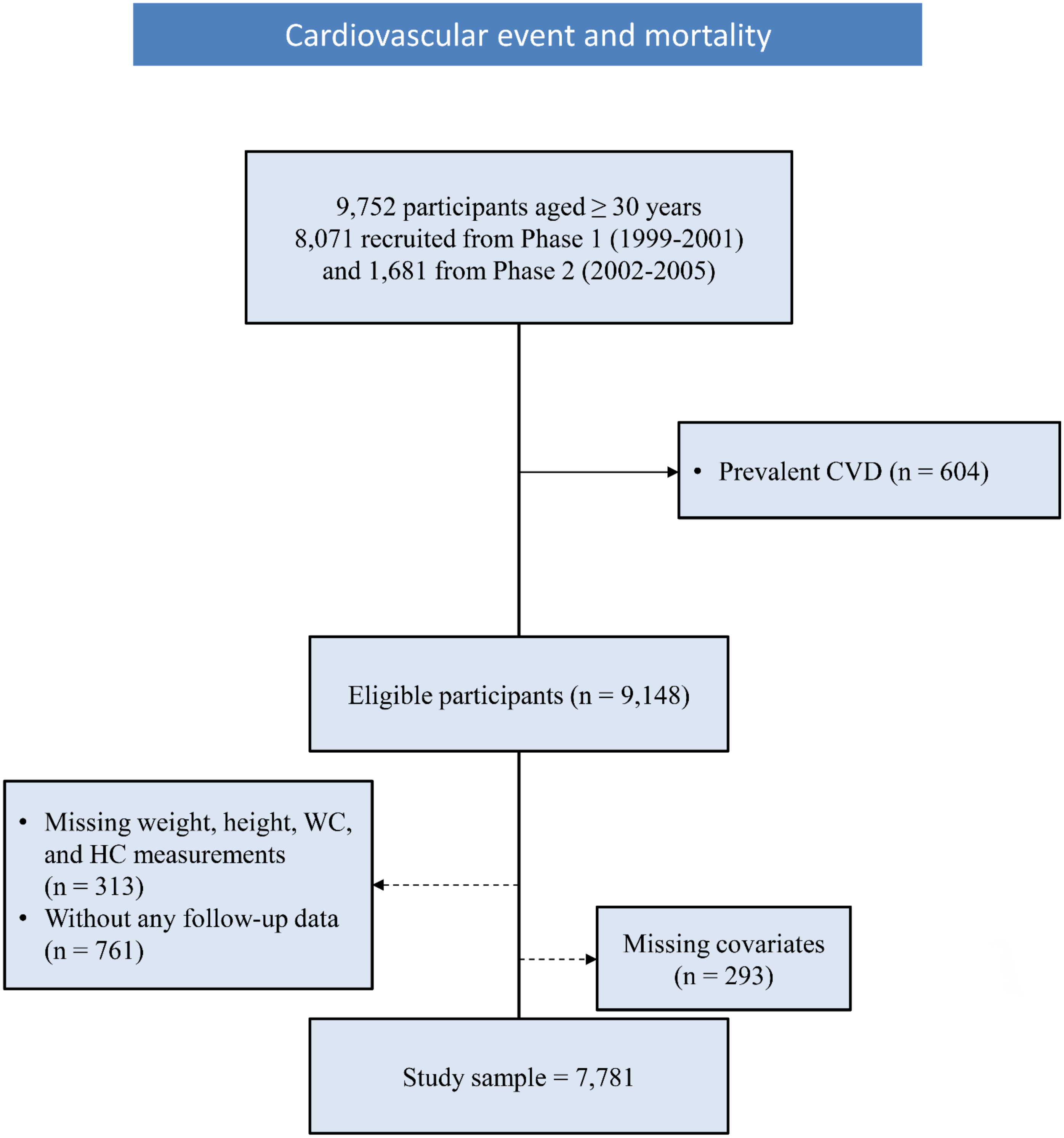

Figure S1. Flowchart of the study population. Abbreviations: CVD, cardiovascular disease; WC, waist circumference; HC, hip circumference.

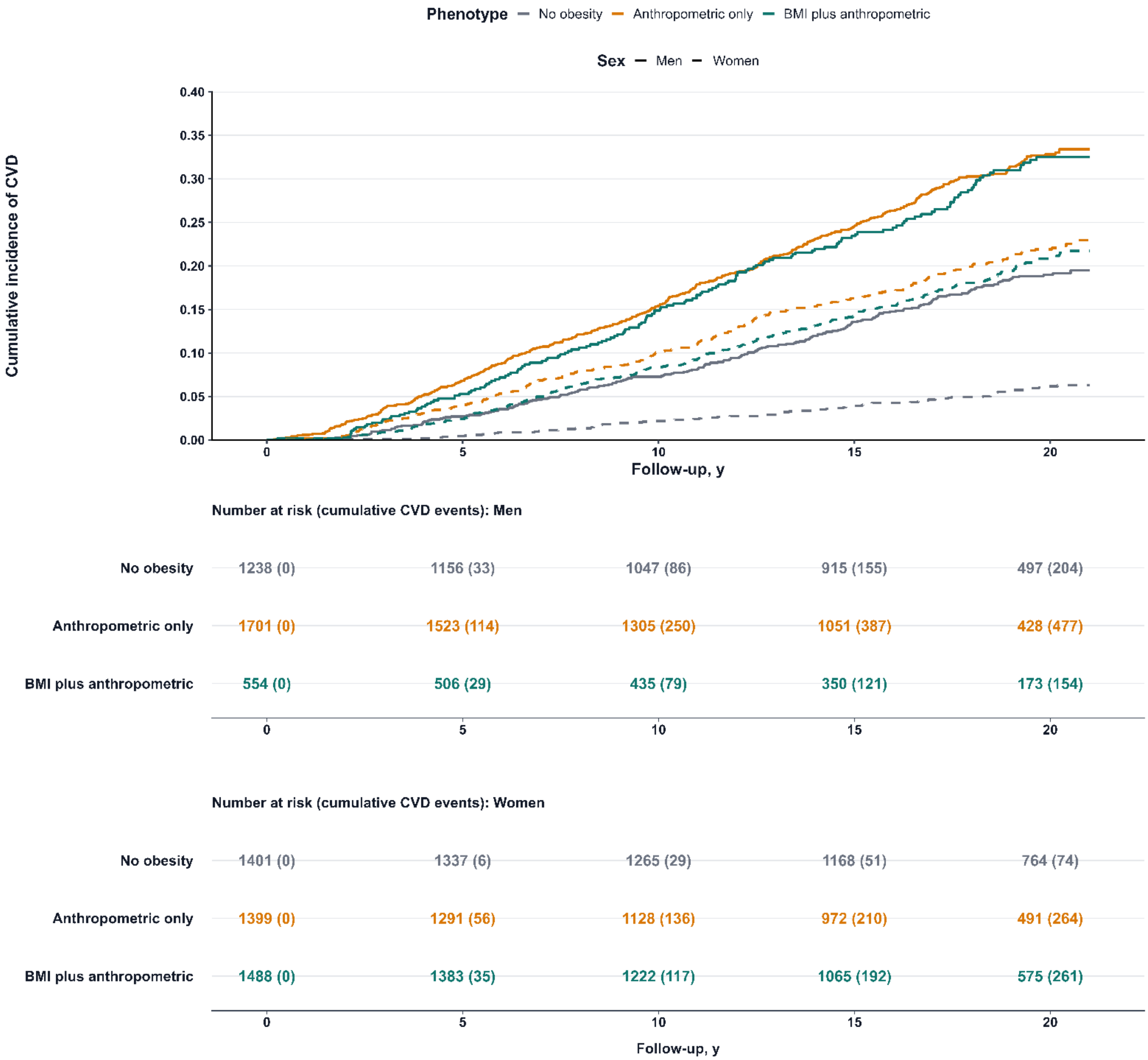

Figure S2. Kaplan-Meier cumulative incidence of CVD by obesity phenotype and sex. Solid lines indicate men; dashed lines indicate women.

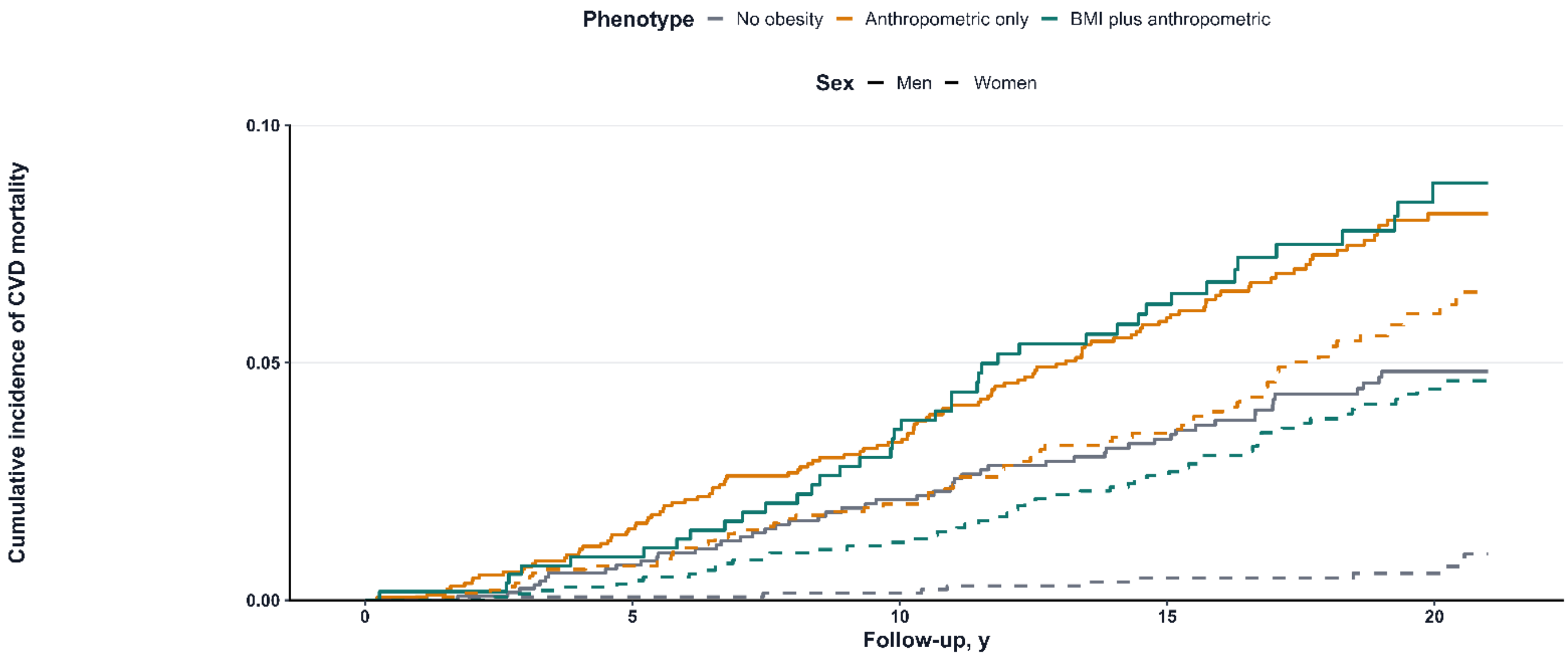

Number at risk (cumulative CVD deaths): Men

	0	5	10	15	20
No obesity	1238 (0)	1179 (9)	1105 (25)	1016 (39)	578 (52)
Anthropometric only	1701 (0)	1609 (25)	1490 (54)	1294 (93)	581 (116)
BMI plus anthropometric	554 (0)	530 (5)	494 (19)	426 (32)	224 (41)

Number at risk (cumulative CVD deaths): Women

	0	5	10	15	20
No obesity	1401 (0)	1342 (1)	1292 (2)	1209 (6)	809 (7)
Anthropometric only	1399 (0)	1335 (10)	1225 (27)	1101 (45)	588 (69)
BMI plus anthropometric	1488 (0)	1411 (5)	1311 (17)	1193 (35)	687 (54)

Follow-up, y

Figure S3. Kaplan-Meier cumulative incidence of CVD mortality by obesity phenotype and sex. Solid lines indicate men; dashed lines indicate women.

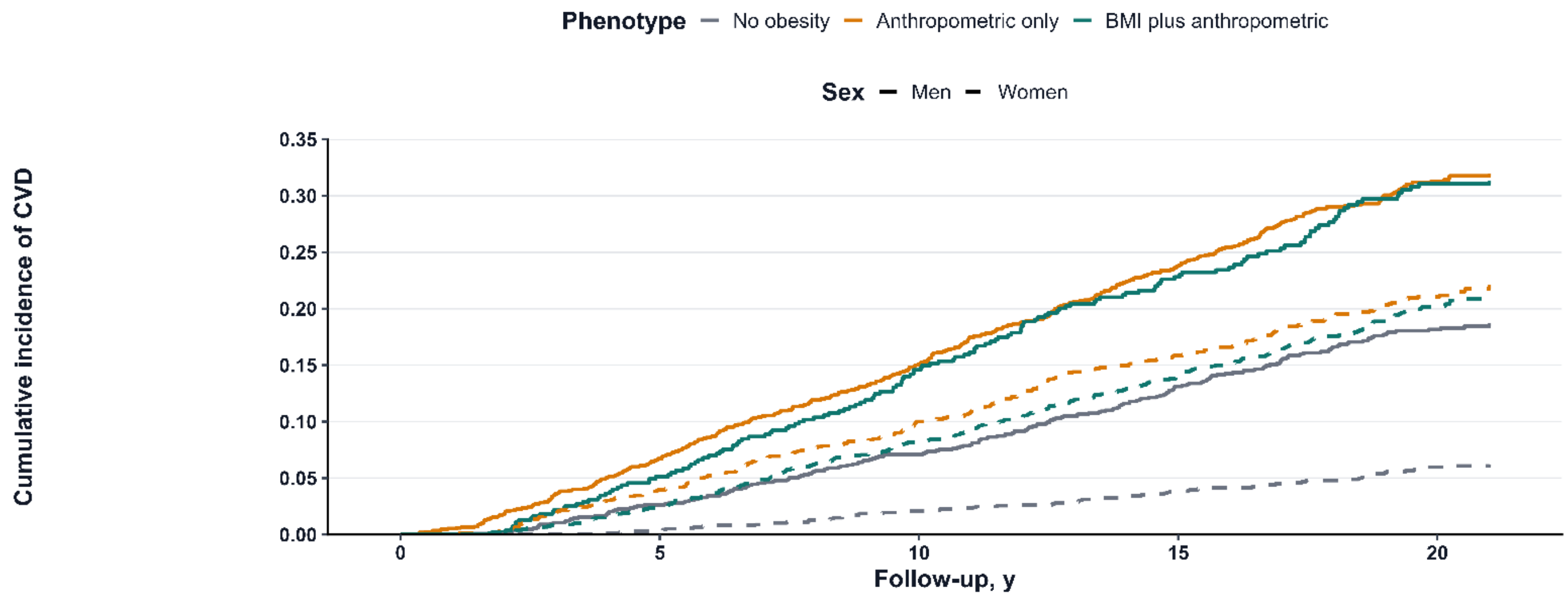

Number at risk (cumulative CVD events): Men

	0	5	10	15	20
No obesity	1238 (0)	1156 (33)	1047 (86)	915 (155)	497 (204)
Anthropometric only	1701 (0)	1523 (114)	1305 (250)	1051 (387)	428 (477)
BMI plus anthropometric	554 (0)	506 (29)	435 (79)	350 (121)	173 (154)

Number at risk (cumulative CVD events): Women

	0	5	10	15	20
No obesity	1401 (0)	1337 (6)	1265 (29)	1168 (51)	764 (74)
Anthropometric only	1399 (0)	1291 (56)	1128 (136)	972 (210)	491 (264)
BMI plus anthropometric	1488 (0)	1383 (35)	1222 (117)	1065 (192)	575 (261)

Follow-up, y

Figure S4. Cumulative incidence function of CVD by obesity phenotype and sex, accounting for non-cardiovascular death as a competing event. Solid lines indicate men; dashed lines indicate women.

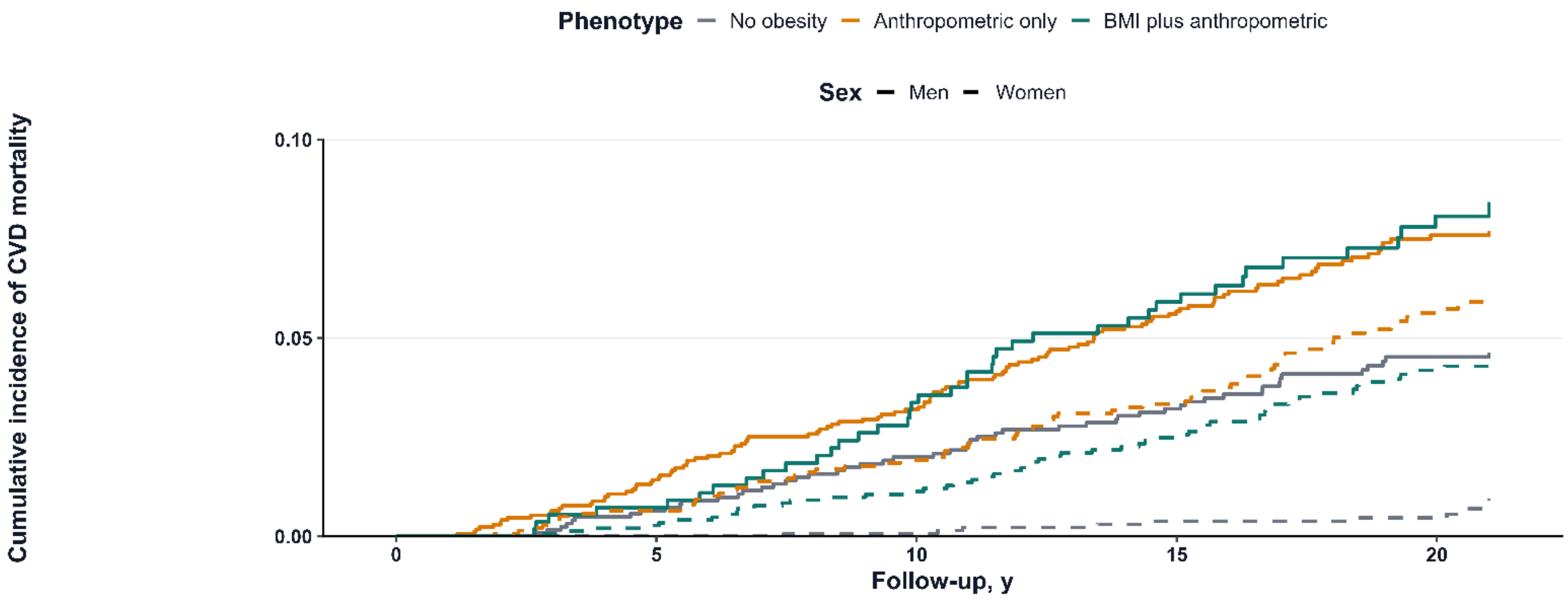

Number at risk (cumulative CVD deaths): Men

	0	5	10	15	20
No obesity	1238 (0)	1179 (9)	1105 (25)	1016 (39)	578 (52)
Anthropometric only	1701 (0)	1609 (25)	1490 (54)	1294 (93)	581 (116)
BMI plus anthropometric	554 (0)	530 (5)	494 (19)	426 (32)	224 (41)

Number at risk (cumulative CVD deaths): Women

	0	5	10	15	20
No obesity	1401 (0)	1342 (1)	1292 (2)	1209 (6)	809 (7)
Anthropometric only	1399 (0)	1335 (10)	1225 (27)	1101 (45)	588 (69)
BMI plus anthropometric	1488 (0)	1411 (5)	1311 (17)	1193 (35)	687 (54)

Follow-up, y

Figure S5. Cumulative incidence function of CVD mortality by obesity phenotype and sex, accounting for non-cardiovascular death as a competing event. Solid lines indicate men; dashed lines indicate women.

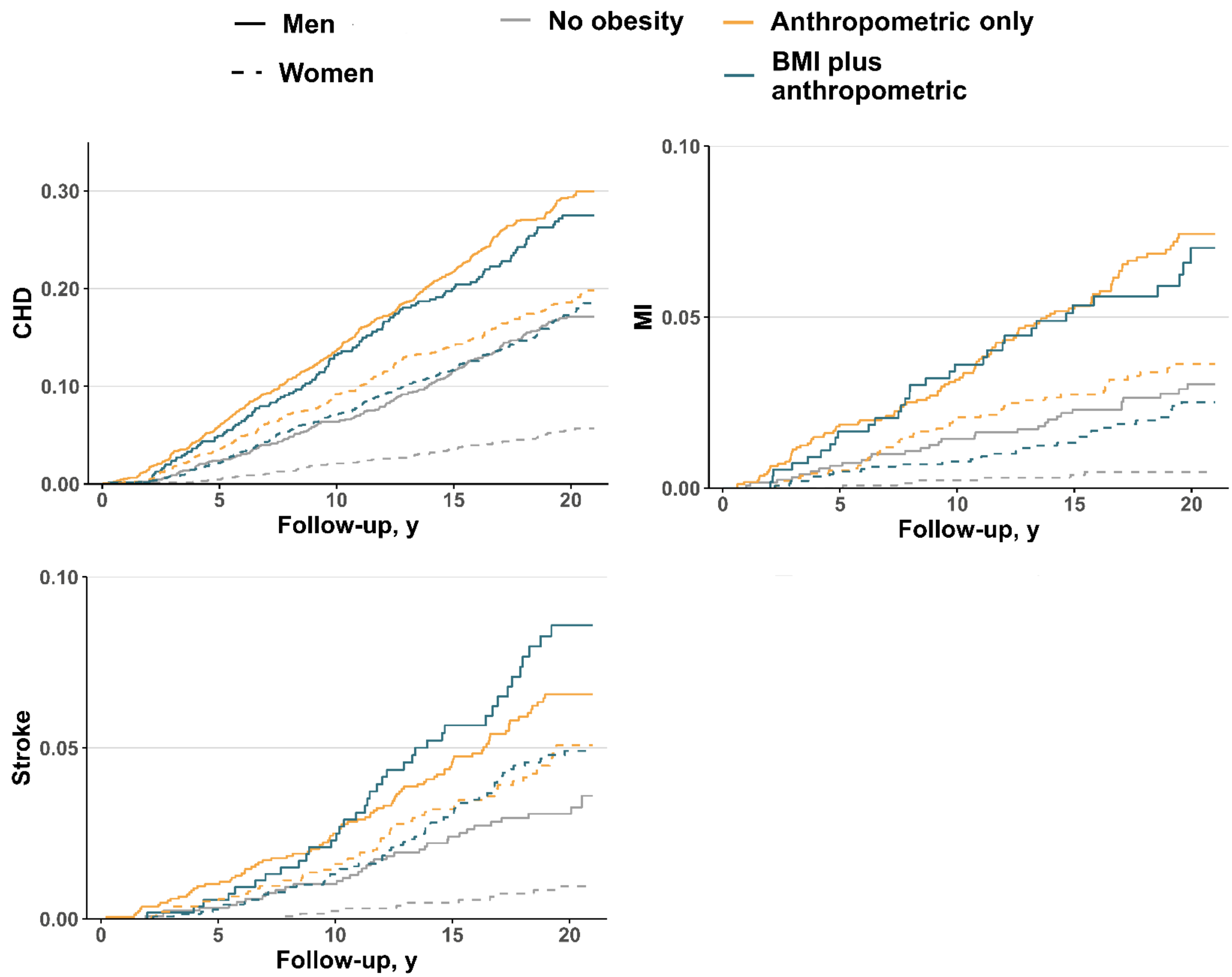

Figure S6. Cumulative incidence of cardiovascular event subtypes across obesity phenotypes, stratified by sex. Solid lines indicate men; dashed lines indicate women.

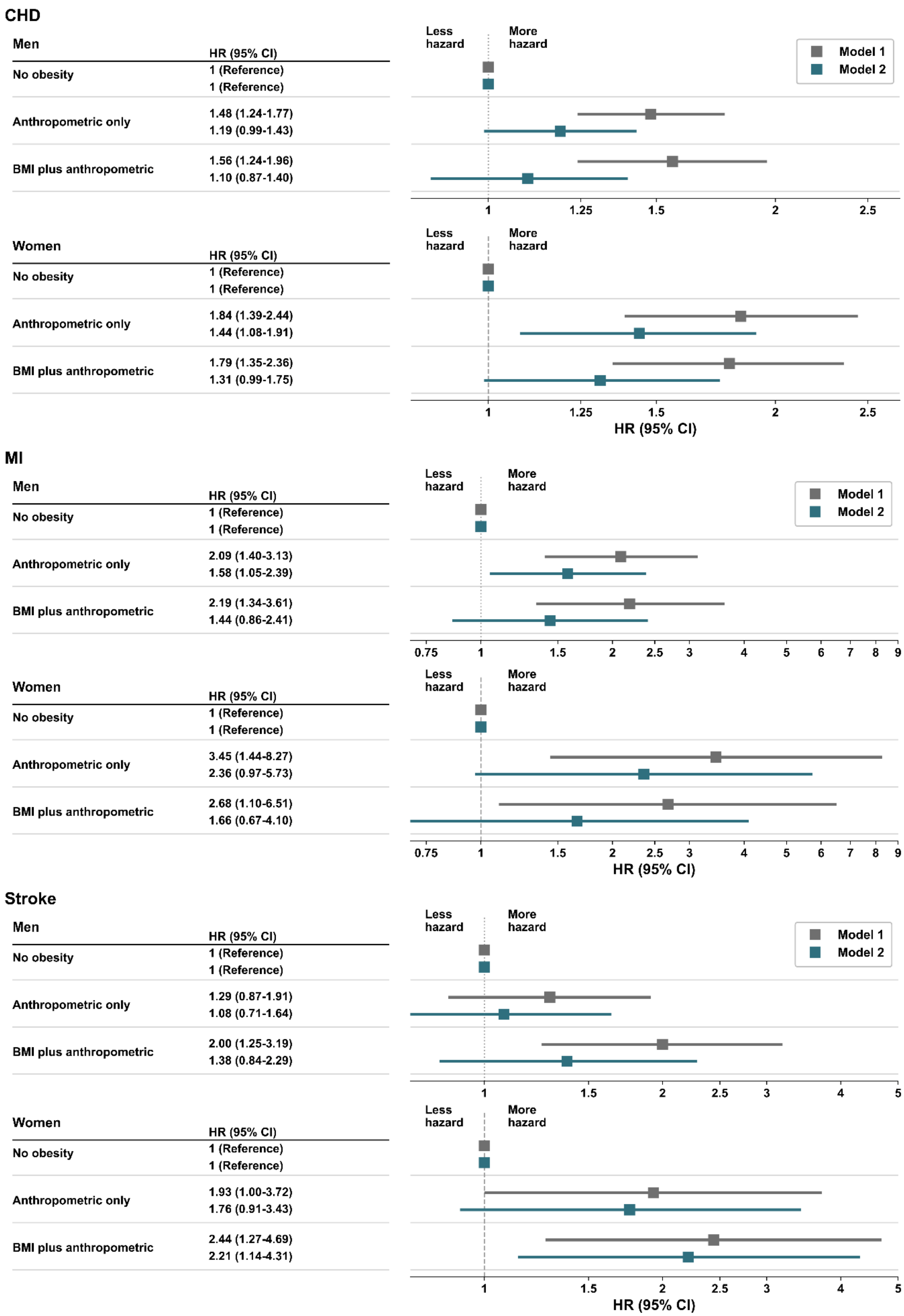

Figure S7. Association of obesity phenotypes with CHD, MI, and stroke, stratified by sex. Hazard ratios (HRs) and 95% confidence intervals (CIs) from Cox proportional hazards models with age as time scale are reported. Model 1 was adjusted for education level, smoking status, and family history of CVD. Model 2 was additionally adjusted for chronic kidney disease, diabetes, hypertension, HDL-C, LDL-C, triglycerides, and lipid-lowering medication use. Abbreviations: CVD, cardiovascular diseases; CHD, coronary heart disease; MI, myocardial infarction; HDL-C, high-density lipoprotein cholesterol; LDL-C, low-density lipoprotein cholesterol; BMI, body mass index.

Table S1. Definitions of obesity

**Traditional Obesity**	Elevated BMI ($\frac{weight}{height^2}$) only	
**New Lancet Commission Obesity**	BMI-plus-anthropometric obesity: Elevated BMI plus at least 1 elevated anthropometric OR BMI ≥40 kg/m^2 (elevated defined below)<br>Anthropometric-only obesity: At least 2 elevated anthropometrics with non-elevated BMI	
**BMI Categories (Based on WHO-recommended cutoffs)**<br>Elevated BMI: ≥30 kg/m^2<br><br>**Traditional BMI Categories**<br>Underweight: <18.5 kg/m^2<br>Normal: BMI 18.5 to <25 kg/m^2<br>Overweight: BMI 25 to <30 kg/m^2<br>Obesity Class I: BMI 30 to <35 kg/m^2<br>Obesity Class II: BMI 35 to <40 kg/m^2<br>Obesity Class III: BMI ≥40 kg/m^2		
**Anthropometric Measurements (Based on sex-specific cut-offs, per *Lancet Commission* supplement)**		
	Men	Women
**Elevated Waist Circumference (WC)**	≥102cm *	≥88 cm*
**Elevated Waist-Height Ratio (WHtR)**	>0.5	>0.5
**Elevated Waist-Hip Ratio (WHR)**	>0.9	>0.85

* Per the Iranian population-specific cut-offs for predicting cardiovascular events, the waist circumference threshold was 95 cm for both sexes.
Abbreviations: BMI, body mass index.

Table S2. Prevalence of obesity by the traditional and new definitions among the TLGS cohort participants (n = 9,429)

U.S.-Based Definition

	Group	Overall	Age, y <45	45-59	≥60
Traditional Definition	Overall	26.3	22.8	32.7	24.6
	Men	15.6	15.2	17.0	14.8
	Women	35.3	29.0	43.9	35.8
New Definition	Overall	67.2	53.7	77.9	81.7
	Men	65.1	54.3	73.5	76.0
	Women	69.0	53.1	80.9	88.3
- BMI plus anthropometric	Overall	26.2	22.8	32.7	24.6
	Men	15.6	15.2	17.0	14.8
	Women	35.2	28.9	43.8	35.8
- Anthropometric only	Overall	41.0	30.9	45.2	57.1
	Men	49.5	39.1	56.5	61.2
	Women	33.8	24.2	37.1	52.5

Iranian-Based Definition

	Group	Overall	Age, y <45	45-59	≥60
Traditional Definition	Overall	26.3	22.8	32.7	24.6
	Men	15.6	15.2	17.0	14.8
	Women	35.3	29.0	43.9	35.8
New Definition	Overall	65.1	50.9	75.8	81.0
	Men	65.3	54.5	73.5	76.0
	Women	65.0	48.0	77.4	86.7
- BMI plus anthropometric	Overall	26.3	22.8	32.7	24.6
	Men	15.6	15.2	17.0	14.8
	Women	35.2	28.9	43.8	35.8
- Anthropometric only	Overall	38.8	28.1	43.1	56.4
	Men	49.7	39.3	56.5	61.2
	Women	29.8	19.1	33.6	50.9

Abbreviations: TLGS, Tehran Lipid and Glucose Study; BMI, body mass index
For the prevalence assessment of the new obesity definition in TLGS, we included 9,429 individuals.

Table S3. Prevalence of obesity under the traditional and new definitions among participants free of prevalent cardiovascular disease at baseline (n = 8,835)

U.S.-Based Definition

	**Group**	**Overall**	**Age, y** **<45**	**45-59**	**≥60**
Traditional Definition	Overall	26.3	22.7	32.7	25.2
	Men	15.5	15.0	16.8	15.1
	Women	35.0	28.9	43.6	36.3
New Definition	Overall	66.1	53.4	77.5	80.9
	Men	64.0	54.0	72.9	75.0
	Women	67.8	52.9	80.6	87.5
- BMI plus anthropometric	Overall	26.2	22.6	32.6	25.2
	Men	15.5	15.0	16.8	15.1
	Women	34.9	28.8	43.5	36.3
- Anthropometric only	Overall	39.9	30.8	44.9	55.7
	Men	48.5	39.1	56.1	59.9
	Women	32.9	24.1	37.1	51.1

Iranian-Based Definition

	**Group**	**Overall**	**Age, y** **<45**	**45-59**	**≥60**
Traditional Definition	Overall	26.3	22.7	32.7	25.2
	Men	15.5	15.0	16.8	15.1
	Women	35.0	28.9	43.6	36.3
New Definition	Overall	63.9	50.6	75.3	80.1
	Men	64.1	54.2	72.9	75.0
	Women	63.7	47.7	76.9	85.8
- BMI plus anthropometric	Overall	26.2	22.6	32.6	25.2
	Men	15.5	15.0	16.8	15.1
	Women	34.9	28.8	43.5	36.3
- Anthropometric only	Overall	37.7	28.0	42.6	55.0
	Men	48.6	39.2	56.1	59.9
	Women	28.8	18.9	33.4	49.5

Abbreviations: TLGS, Tehran Lipid and Glucose Study; BMI, body mass index
For the prevalence assessment of the new obesity definition in TLGS, we included 9,429 individuals.

Table S4. Association of the new definition of obesity with cardiovascular event subtypes, stratified by sex

Outcome	Sex	Groups	Case/total	Model 1			Model 2		
				HR (95% CI)	*P* value	*P* interaction	HR (95% CI)	*P* value	*P* interaction
CHD	Men	No obesity	181/1,238	1.00 (Reference)	…	**0.02**	1.00 (Reference)	…	**0.05**
		With obesity	555/2,255	**1.50 (1.27-1.78)**	**<0.001**		1.17 (0.98-1.40)	0.08	
	Women	No obesity	68/1,401	1.00 (Reference)	…		1.00 (Reference)	…	
		With obesity	450/2,887	**1.81 (1.39-2.36)**	**<0.001**		**1.37 (1.05-1.80)**	**0.02**	
MI	Men	No obesity	32/1,238	1.00 (Reference)	…	0.14	1.00 (Reference)	…	0.22
		With obesity	135/2,255	**2.12 (1.43-3.13)**	**<0.001**		**1.55 (1.03-2.32)**	**0.03**	
	Women	No obesity	6/1,401	1.00 (Reference)	…		1.00 (Reference)	…	
		With obesity	73/2,887	**3.06 (1.31-7.15)**	**0.01**		2.16 (0.91-5.13)	0.08	
Stroke	Men	No obesity	35/1,238	1.00 (Reference)	…	0.17	1.00 (Reference)	…	0.22
		With obesity	128/2,255	1.44 (0.98-2.10)	0.06		1.14 (0.76-1.71)	0.52	
	Women	No obesity	11/1,401	1.00 (Reference)	…		1.00 (Reference)	…	
		With obesity	116/2,887	**2.17 (1.16-4.06)**	**0.02**		**1.97 (1.04-3.72)**	**0.04**	

Bold values indicate statistical significance (P <0.05)
Hazard ratios (HRs) and 95% confidence intervals (CIs) from Cox proportional hazards models with age as time scale are reported.
Model 1 was adjusted for education level, smoking status, and family history of CVD.
Model 2 was additionally adjusted for chronic kidney disease, diabetes, hypertension, HDL-C, LDL-C, triglycerides, and lipid-lowering medication use.
Abbreviation: CVD: cardiovascular disease, CHD: coronary heart disease, MI: myocardial infarction; HR, hazard ratio; CI, confidence interval; HDL-C, high-density lipoprotein cholesterol; LDL-C, low-density lipoprotein cholesterol.

Table S5. Association of the new obesity definition with cardiovascular events using Iranian waist circumference cutoffs* for elevated WC, stratified by sex

Outcome	Sex	Groups	Case/total	Model 1			Model 2		
				HR (95% CI)	*P* value	*P* interaction	HR (95% CI)	*P* value	*P* interaction
**CVD**	Men	No obesity	206/1,234	1.00 (Reference)	…	0.007	1.00 (Reference)	…	0.02
		With obesity	635/2,259	1.48 (1.26-1.73)	<0.001		1.16 (0.98-1.37)	0.09	
	Women	No obesity	92/1,576	1.00 (Reference)	…		1.00 (Reference)	…	
		With obesity	517/2,712	1.84 (1.46-2.31)	<0.001		1.45 (1.15-1.83)	0.002	
**CVD mortality**	Men	No obesity	52/1,234	1.00 (Reference)	…	0.008	1.00 (Reference)	…	0.02
		With obesity	157/2,259	1.26 (0.92-1.73)	0.15		1.08 (0.76-1.52)	0.68	
	Women	No obesity	11/1,576	1.00 (Reference)	…		1.00 (Reference)	…	
		With obesity	124/2,712	2.84 (1.51-5.32)	0.001		2.23 (1.17-4.23)	0.02	
**CHD**	Men	No obesity	181/1,234	1.00 (Reference)	…	0.02	1.00 (Reference)	…	0.06
		With obesity	555/2,259	1.49 (1.26-1.77)	<0.001		1.16 (0.97-1.39)	0.09	
	Women	No obesity	83/1,576	1.00 (Reference)	…		1.00 (Reference)	…	
		With obesity	435/2,712	1.76 (1.38-2.24)	<0.001		1.35 (1.05-1.73)	0.02	
**MI**	Men	No obesity	32/1,234	1.00 (Reference)	…	0.10	1.00 (Reference)	…	0.16
		With obesity	135/2,259	2.11 (1.43-3.12)	<0.001		1.54 (1.03-2.31)	0.04	
	Women	No obesity	7/1,576	1.00 (Reference)	…		1.00 (Reference)	…	
		With obesity	72/2,712	3.19 (1.45-7.05)	0.004		2.29 (1.02-5.13)	0.04	
**Stroke**	Men	No obesity	35/1,234	1.00 (Reference)	…	0.14	1.00 (Reference)	…	0.19
		With obesity	128/2,259	1.43 (0.98-2.09)	0.06		1.14 (0.76-1.71)	0.53	
	Women	No obesity	13/1,576	1.00 (Reference)	…		1.00 (Reference)	…	
		With obesity	114/2,712	**2.17 (1.21-3.89)**	**0.01**		**1.98 (1.10-3.59)**	**0.02**	

* ≥95 cm for both sexes
Bold values indicate statistical significance (*P* <0.05)
Hazard ratios (HRs) and 95% confidence intervals (CIs) from Cox's proportional hazards models with age as time scale.
Model 1 was adjusted for education level, smoking status, and family history of CVD. Model 2 was additionally adjusted for chronic kidney disease, diabetes, hypertension, HDL-C, LDL-C, triglycerides, and lipid-lowering medication use.
Abbreviations: WC, waist circumference; CVD, cardiovascular disease; CHD, coronary heart disease; MI, myocardial infarction; HR, hazard ratio; CI, confidence interval; HDL-C, high-density lipoprotein cholesterol; LDL-C, low-density lipoprotein cholesterol.

Table S6. Association of the new definition of obesity with a composite of nonfatal MI and nonfatal stroke stratified by sex

	Men					Women				
	Case/total	Model 1		Model 2		Case/total	Model 1		Model 2	
		HR (95% CI)	*P* value	HR (95% CI)	*P* value		HR (95% CI)	*P* value	HR (95% CI)	*P* value
**Composite of nonfatal MI and nonfatal stroke**										
- No obesity	55/1,238	1.00 (Reference)	…	1.00 (Reference)	…	16/1,401	1.00 (Reference)	…	1.00 (Reference)	…
- With obesity	214/2,255	**1.78 (1.31-2.40)**	**<0.001**	1.33 (0.97-1.83)	0.074	145/2,887	**2.07 (1.22-3.50)**	**0.007**	**1.78 (1.04-3.05)**	**0.035**
- No obesity	55/1,238	1.00 (Reference)	…	1.00 (Reference)	…	16/1,401	1.00 (Reference)	…	1.00 (Reference)	…
- Anthropometric-only obesity	153/1,701	**1.64 (1.20-2.24)**	**0.002**	1.28 (0.93-1.77)	0.133	75/1,399	**1.99 (1.14-3.45)**	**0.015**	1.72 (0.98-3.02)	0.057
- BMI-plus-anthropometric obesity	61/554	**2.27 (1.57-3.29)**	**<0.001**	**1.53 (1.03-2.26)**	**0.033**	70/1,488	**2.15 (1.24-3.74)**	**0.007**	**1.84 (1.05-3.24)**	**0.034**

The interaction between the new obesity definition and sex in relation to the composite outcome of nonfatal MI and nonfatal stroke was not statistically significant. The P values for interaction were 0.169 in Model 1 and 0.253 in Model 2.

Bold values indicate statistical significance ($P<0.05$)

Hazard ratios (HRs) and 95% confidence intervals (CIs) from Cox proportional hazards models with age as time scale are reported.

Model 1 was adjusted for education level, smoking status, and family history of CVD.

Model 2 was additionally adjusted for chronic kidney disease, diabetes, hypertension, HDL-C, LDL-C, triglycerides, and lipid-lowering medication use.

Abbreviation: CVD, cardiovascular disease; MI, myocardial infarction; HR, hazard ratio; CI, confidence interval; HDL-C, high-density lipoprotein cholesterol; LDL-C, low-density lipoprotein cholesterol.

Table 7. Associations of obesity phenotypes with CVD mortality after excluding events occurring within the first 5 years of follow-up*

Phenotypes	Men			Women		
	Events (%)	HR (95% CI)		Events (%)	HR (95% CI)	
		Model 1	Model 2		Model 1	Model 2
- No obesity	43 (3.5)	1.00 (Reference)	1.00 (Reference)	8 (0.6)	1.00 (Reference)	1.00 (Reference)
- With obesity	127 (5.7)	1.24 (0.87-1.77)	1.08 (0.74-1.58)	111 (3.9)	**2.77 (1.34-5.75)**	**2.22 (1.06-4.65)**
- No obesity	43 (3.5)	1.00 (Reference)	1.00 (Reference)	8 (0.6)	1.00 (Reference)	1.00 (Reference)
- Anthropometric-only obesity	91 (5.4)	1.12 (0.78-1.62)	1.02 (0.69-1.50)	61 (4.4)	**2.64 (1.25-5.60)**	**2.19 (1.02-4.69)**
- BMI-plus-anthropometric obesity	36 (6.6)	**1.77 (1.12-2.78)**	1.39 (0.85-2.27)	50 (3.4)	**2.94 (1.38-6.28)**	**2.25 (1.04-4.87)**

* 55 CVD mortality events occurred in the first 5 years of follow-up.
Hazard ratios (HRs) and 95% confidence intervals (CIs) from Cox proportional hazards models with age as time scale are reported.
Model 1 was adjusted for education level, smoking status, and family history of CVD. Model 2 was additionally adjusted for chronic kidney disease, diabetes, hypertension, HDL-C, LDL-C, triglycerides, and lipid-lowering medication use.
Abbreviations: CVD, cardiovascular disease; HR, hazard ratio; CI, confidence interval; HDL-C, high-density lipoprotein cholesterol; LDL-C, low-density lipoprotein cholesterol.

Table S8. Association of the new definition of obesity with CVD and CVD mortality stratified by sex, with adjustment for LDL-C estimated using the modified Friedewald formula

	Men		Women	
	HR (95% CI)	*P* value	HR (95% CI)	*P* value
**CVD**				
- No obesity	1.00 (Reference)	…	1.00 (Reference)	…
- With obesity	1.16 (0.98-1.37)	0.075	**1.49 (1.16-1.92)**	**0.002**
- No obesity	1.00 (Reference)	…	1.00 (Reference)	…
- Anthropometric-only obesity	1.17 (0.99-1.39)	0.072	**1.50 (1.14-1.96)**	**0.003**
- BMI-plus-anthropometric obesity	1.14 (0.91-1.42)	0.251	**1.48 (1.13-1.94)**	**0.004**
**CVD mortality**				
- No obesity	1.00 (Reference)	…	1.00 (Reference)	…
- With obesity	1.08 (0.76-1.52)	0.678	**2.33 (1.16-4.71)**	**0.018**
- No obesity	1.00 (Reference)	…	1.00 (Reference)	…
- Anthropometric-only obesity	1.05 (0.74-1.48)	0.805	**2.42 (1.18-4.96)**	**0.016**
- BMI-plus-anthropometric obesity	1.21 (0.77-1.90)	0.400	**2.23 (1.07-4.64)**	**0.031**

Bold values indicate statistical significance at P <0.05.

Hazard ratios (HRs) and 95% confidence intervals (CIs) were estimated using Cox proportional hazards models with age as the time scale. Models were adjusted for education level, smoking status, family history of CVD, chronic kidney disease, diabetes, hypertension, LDL-C (estimated using the modified Friedewald formula), HDL-C, triglycerides, and lipid-lowering medication use.

Abbreviations: CVD, cardiovascular disease; HR, hazard ratio; CI, confidence interval; BMI, body mass index; LDL-C, low-density lipoprotein cholesterol; HDL-C, high-density lipoprotein cholesterol.

Table S9. Association of the traditional definition of obesity with CVD and CVD mortality stratified by sex

	Men				Women			
	Model 1		Model 2		Model 1		Model 2	
	HR (95% CI)	*P* value	HR (95% CI)	*P* value	HR (95% CI)	*P* value	HR (95% CI)	*P* value
**CVD**								
- No obesity	1.00 (Reference)	…	1.00 (Reference)	…	1.00 (Reference)	…	1.00 (Reference)	…
- With obesity	**1.26 (1.06-1.51)**	**0.009**	1.01 (0.85-1.21)	0.889	**1.24 (1.05-1.46)**	**0.009**	1.10 (0.93-1.29)	0.276
**CVD mortality**								
- No obesity	1.00 (Reference)	…	1.00 (Reference)	…	1.00 (Reference)	…	1.00 (Reference)	…
- With obesity	**1.44 (1.02-2.04)**	**0.039**	1.17 (0.82-1.68)	0.377	1.24 (0.87-1.76)	0.233	1.06 (0.74-1.51)	0.753

Bold values indicate statistical significance (P <0.05)
Hazard ratios (HRs) and 95% confidence intervals (CIs) from Cox proportional hazards models with age as time scale are reported.
Model 1 was adjusted for education level, smoking status, and family history of CVD.
Model 2 was additionally adjusted for chronic kidney disease, diabetes, hypertension, HDL-C, LDL-C, triglycerides, and lipid-lowering medication use.
Abbreviation: CVD: cardiovascular disease; HR, hazard ratio; CI, confidence interval; HDL-C, high-density lipoprotein cholesterol; LDL-C, low-density lipoprotein cholesterol.

Table S10. Sex-stratified associations of the new definition of obesity, modeled as a time-varying covariate, with cardiovascular event subtypes

CHD	Men				Women			
	Model 1		Model 2		Model 1		Model 2	
	HR (95% CI)	*P* value	HR (95% CI)	*P* value	HR (95% CI)	*P* value	HR (95% CI)	*P* value
Obesity-Free	1.00 (Reference)	…	1.00 (Reference)	…	1.00 (Reference)	…	1.00 (Reference)	…
With Obesity	**1.54 (1.23-1.94)**	**<0.001**	**1.28 (1.01-1.62)**	**0.03**	**2.51 (1.59-3.94)**	**<0.001**	**1.91 (1.21-3.03)**	**0.006**
No Obesity	1.00 (Reference)	…	1.00 (Reference)	…	1.00 (Reference)	…	1.00 (Reference)	…
Anthropometric-only Obesity	**1.51 (1.19-1.90)**	**<0.001**	**1.29 (1.02-1.64)**	**0.04**	**2.42 (1.52-3.85)**	**<0.001**	**1.98 (1.24-3.17)**	**0.004**
BMI plus anthropometric Obesity	**1.67 (1.28-2.18)**	**<0.001**	1.25 (0.95-1.64)	0.11	**2.58 (1.63-4.09)**	**<0.001**	**1.86 (1.16-2.97)**	**0.009**

MI	Men				Women			
	Model 1		Model 2		Model 1		Model 2	
	HR (95% CI)	*P* value	HR (95% CI)	*P* value	HR (95% CI)	*P* value	HR (95% CI)	*P* value
Obesity-Free	1.00 (Reference)	…	1.00 (Reference)	…	1.00 (Reference)	…	1.00 (Reference)	…
With Obesity	**2.67 (1.51-4.71)**	**<0.001**	**2.25 (1.25-4.04)**	**0.01**	1.79 (0.61-5.29)	0.29	1.38 (0.47-4.07)	0.56
No Obesity	1.00 (Reference)	…	1.00 (Reference)	…	1.00 (Reference)	…	1.00 (Reference)	…
Anthropometric-only Obesity	**2.47 (1.38-4.40)**	**0.002**	**2.15 (1.19-3.89)**	**0.01**	1.63 (0.52-5.09)	0.40	1.36 (0.42-4.39)	0.60
BMI plus anthropometric Obesity	**3.31 (1.78-6.14)**	**<0.001**	**2.59 (1.36-4.93)**	**0.004**	1.95 (0.66-5.74)	0.22	1.44 (0.46-4.49)	0.53

Stroke	Men				Women			
	Model 1		Model 2		Model 1		Model 2	
	HR (95% CI)	*P* value	HR (95% CI)	*P* value	HR (95% CI)	*P* value	HR (95% CI)	*P* value
Obesity-Free	1.00 (Reference)	…	1.00 (Reference)	…	1.00 (Reference)	…	1.00 (Reference)	…
With Obesity	1.30 (0.79-2.14)	0.31	1.00 (0.60-1.67)	0.98	**4.01 (1.01-16.02)**	**0.04**	3.58 (0.89-14.38)	0.07
No Obesity	1.00 (Reference)	…	1.00 (Reference)	…	1.00 (Reference)	…	1.00 (Reference)	…
Anthropometric-only Obesity	1.18 (0.71-1.97)	0.53	0.97 (0.58-1.62)	0.89	3.56 (0.87-14.60)	0.07	3.28 (0.80-13.52)	0.10
BMI plus anthropometric Obesity	**1.77 (1.00-3.12)**	**0.04**	1.16 (0.64-2.08)	0.62	**4.53 (1.14-18.03)**	**0.03**	3.96 (0.99-15.88)	0.05

Bold values indicate statistical significance ($P < 0.05$)
Hazard ratios and 95% confidence intervals were estimated using time-varying Cox proportional hazards models, in which both the primary exposure and covariates were modeled as time-dependent variables (except for education level and family history of CVD).
Model 1 was adjusted for age, education level, smoking status, and family history of CVD.
Model 2 was additionally adjusted for chronic kidney disease, diabetes, hypertension, and hypercholesterolemia.
Abbreviation: CVD, cardiovascular disease; CHD, coronary heart disease; MI, myocardial infarction; HR, hazard ratio; CI, confidence interval.

Table S11. Availability of variables included in the time-varying analyses across seven study visits

Variable	Mean observed visits, mean (SD)	Median observed visits	Total observed measurements
Time since baseline	4.96 (2.01)	6	38,628
Smoking status	4.91 (2.01)	6	38,200
Diabetes	4.90 (2.01)	6	38,149
Hypertension	4.90 (2.01)	6	38,116
Chronic kidney disease	4.93 (2.01)	6	38,380
Hypercholesterolemia	4.92 (2.01)	6	38,272
Obesity phenotype grouping	4.80 (2.04)	5	37,369

Values represent the number of observed, non-missing measurements available across seven study visits among 7,781 participants. The mean and median observed visits indicate the number of visits per participant with available data for each variable. Total observed measurements represent the cumulative number of non-missing observations across all participants and visits. No carry-forward procedure, last-observation-carried-forward method, or other imputation approach was applied.
